# An open-source framework for end-to-end analysis of electronic health record data

**DOI:** 10.1038/s41591-024-03214-0

**Published:** 2024-09-12

**Authors:** Lukas Heumos, Philipp Ehmele, Tim Treis, Julius Upmeier zu Belzen, Eljas Roellin, Lilly May, Altana Namsaraeva, Nastassya Horlava, Vladimir A. Shitov, Xinyue Zhang, Luke Zappia, Rainer Knoll, Niklas J. Lang, Leon Hetzel, Isaac Virshup, Lisa Sikkema, Fabiola Curion, Roland Eils, Herbert B. Schiller, Anne Hilgendorff, Fabian J. Theis

**Affiliations:** 1 Institute of Computational Biology, Helmholtz Munich, Munich, Germany; 2 Institute of Lung Health and Immunity and Comprehensive Pneumology Center with the CPC-M bioArchive; Helmholtz Zentrum Munich; member of the German Center for Lung Research (DZL), Munich, Germany; 3 TUM School of Life Sciences Weihenstephan, Technical University of Munich, Munich, Germany; 4 Health Data Science Unit, Heidelberg University and BioQuant, Heidelberg, Germany; 5 Department of Mathematics, School of Computation, Information and Technology, Technical University of Munich, Munich, Germany; 6 Konrad Zuse School of Excellence in Learning and Intelligent Systems (ELIZA), Darmstadt, Germany; 7 Systems Medicine, Deutsches Zentrum für Neurodegenerative Erkrankungen (DZNE), Bonn, Germany; 8 Center for Digital Health, Berlin Institute of Health (BIH) at Charité – Universitätsmedizin Berlin, Berlin, Germany; 9 Research Unit, Precision Regenerative Medicine (PRM), Helmholtz Munich, Munich, Germany; 10 Center for Comprehensive Developmental Care (CDeCLMU) at the Social Pediatric Center, Dr. von Hauner Children’s Hospital, LMU Hospital, Ludwig Maximilian University, Munich, Germany

**Keywords:** Epidemiology, Translational research

## Abstract

With progressive digitalization of healthcare systems worldwide, large-scale collection of electronic health records (EHRs) has become commonplace. However, an extensible framework for comprehensive exploratory analysis that accounts for data heterogeneity is missing. Here we introduce ehrapy, a modular open-source Python framework designed for exploratory analysis of heterogeneous epidemiology and EHR data. ehrapy incorporates a series of analytical steps, from data extraction and quality control to the generation of low-dimensional representations. Complemented by rich statistical modules, ehrapy facilitates associating patients with disease states, differential comparison between patient clusters, survival analysis, trajectory inference, causal inference and more. Leveraging ontologies, ehrapy further enables data sharing and training EHR deep learning models, paving the way for foundational models in biomedical research. We demonstrate ehrapy’s features in six distinct examples. We applied ehrapy to stratify patients affected by unspecified pneumonia into finer-grained phenotypes. Furthermore, we reveal biomarkers for significant differences in survival among these groups. Additionally, we quantify medication-class effects of pneumonia medications on length of stay. We further leveraged ehrapy to analyze cardiovascular risks across different data modalities. We reconstructed disease state trajectories in patients with severe acute respiratory syndrome coronavirus 2 (SARS-CoV-2) based on imaging data. Finally, we conducted a case study to demonstrate how ehrapy can detect and mitigate biases in EHR data. ehrapy, thus, provides a framework that we envision will standardize analysis pipelines on EHR data and serve as a cornerstone for the community.

## Abstract (web summary)

Incorporating a series of analytical steps, from data extraction and quality control to the generation of low-dimensional representations and to longitudinal analyses, an open-source software is proposed to standardize current electronic health record data processing and analysis pipelines.

## Main

Electronic health records (EHRs) are becoming increasingly common due to standardized data collection^1^ and digitalization in healthcare institutions. EHRs collected at medical care sites serve as efficient storage and sharing units of health information^2^, enabling the informed treatment of individuals using the patient’s complete history^3^. Routinely collected EHR data are approaching genomic-scale size and complexity^4^, posing challenges in extracting information without quantitative analysis methods. The application of such approaches to EHR databases^1,5–9^ has enabled the prediction and classification of diseases^10,11^, study of population health^12^, determination of optimal treatment policies^13,14^, simulation of clinical trials^15^ and stratification of patients^16^.

However, current EHR datasets suffer from serious limitations, such as data collection issues, inconsistencies and lack of data diversity. EHR data collection and sharing problems often arise due to non-standardized formats, with disparate systems using exchange protocols, such as Health Level Seven International (HL7) and Fast Healthcare Interoperability Resources (FHIR)^17^. In addition, EHR data are stored in various on-disk formats, including, but not limited to, relational databases and CSV, XML and JSON formats. These variations pose challenges with respect to data retrieval, scalability, interoperability and data sharing.

Beyond format variability, inherent biases of the collected data can compromise the validity of findings. Selection bias stemming from non-representative sample composition can lead to skewed inferences about disease prevalence or treatment efficacy^18,19^. Filtering bias arises through inconsistent criteria for data inclusion, obscuring true variable relationships^20^. Surveillance bias exaggerates associations between exposure and outcomes due to differential monitoring frequencies^21^. EHR data are further prone to missing data^22,23^, which can be broadly classified into three categories: missing completely at random (MCAR), where missingness is unrelated to the data; missing at random (MAR), where missingness depends on observed data; and missing not at random (MNAR), where missingness depends on unobserved data^22,23^. Information and coding biases, related to inaccuracies in data recording or coding inconsistencies, respectively, can lead to misclassification and unreliable research conclusions^24,25^. Data may even contradict itself, such as when measurements were reported for deceased patients^26,27^. Technical variation and differing data collection standards lead to distribution differences and inconsistencies in representation and semantics across EHR datasets^28,29^. Attrition and confounding biases, resulting from differential patient dropout rates or unaccounted external variable effects, can significantly skew study outcomes^30–32^. The diversity of EHR data that comprise demographics, laboratory results, vital signs, diagnoses, medications, x-rays, written notes and even omics measurements amplifies all the aforementioned issues.

Addressing these challenges requires rigorous study design, careful data pre-processing and continuous bias evaluation through exploratory data analysis. Several EHR data pre-processing and analysis workflows were previously developed^4,33–37^, but none of them enables the analysis of heterogeneous data, provides in-depth documentation, is available as a software package or allows for exploratory visual analysis. Current EHR analysis pipelines, therefore, differ considerably in their approaches and are often commercial, vendor-specific solutions^38^. This is in contrast to strategies using community standards for the analysis of omics data, such as Bioconductor^39^ or scverse^40^. As a result, EHR data frequently remain underexplored and are commonly investigated only for a particular research question^41^. Even in such cases, EHR data are then frequently input into machine learning models with serious data quality issues that greatly impact prediction performance and generalizability^42^.

To address this lack of analysis tooling, we developed the EHR Analysis in Python framework, ehrapy, which enables exploratory analysis of diverse EHR datasets. The ehrapy package is purpose-built to organize, analyze, visualize and statistically compare complex EHR data. ehrapy can be applied to datasets of different data types, sizes, diseases and origins. To demonstrate this versatility, we applied ehrapy to datasets obtained from EHR and population-based studies. Using the Pediatric Intensive Care (PIC) EHR database^43^, we stratified patients diagnosed with ‘unspecified pneumonia’ into distinct clinically relevant groups, extracted clinical indicators of pneumonia through statistical analysis and quantified medication-class effects on length of stay (LOS) with causal inference. Using the UK Biobank^44^ (UKB), a population-scale cohort comprising over 500,000 participants from the United Kingdom, we employed ehrapy to explore cardiovascular risk factors using clinical predictors, metabolomics, genomics and retinal imaging-derived features. Additionally, we performed image analysis to project disease progression through fate mapping in patients affected by coronavirus disease 2019 (COVID-19) using chest x-rays. Finally, we demonstrate how exploratory analysis with ehrapy unveils and mitigates biases in over 100,000 visits by patients with diabetes across 130 US hospitals. We provide online links to additional use cases that demonstrate ehrapy’s usage with further datasets, including MIMIC-II (ref. ^45^), and for various medical conditions, such as patients subject to indwelling arterial catheter usage. ehrapy is compatible with any EHR dataset that can be transformed into vectors and is accessible as a user-friendly open-source software package hosted at https://github.com/theislab/ehrapy and installable from PyPI. It comes with comprehensive documentation, tutorials and further examples, all available at https://ehrapy.readthedocs.io.

## Results

### ehrapy: a framework for exploratory EHR data analysis

The foundation of ehrapy is a robust and scalable data storage backend that is combined with a series of pre-processing and analysis modules. In ehrapy, EHR data are organized as a data matrix where observations are individual patient visits (or patients, in the absence of follow-up visits), and variables represent all measured quantities (Methods). These data matrices are stored together with metadata of observations and variables. By leveraging the AnnData (annotated data) data structure that implements this design, ehrapy builds upon established standards and is compatible with analysis and visualization functions provided by the omics scverse^40^ ecosystem. Readers are also available in R, Julia and Javascript^46^. We additionally provide a dataset module with more than 20 public loadable EHR datasets in AnnData format to kickstart analysis and development with ehrapy.

For standardized analysis of EHR data, it is crucial that these data are encoded and stored in consistent, reusable formats. Thus, ehrapy requires that input data are organized in structured vectors. Readers for common formats, such as CSV, OMOP^47^ or SQL databases, are available in ehrapy. Data loaded into AnnData objects can be mapped against several hierarchical ontologies^48–51^ (Methods). Clinical keywords of free text notes can be automatically extracted (Methods).

**Fig. 1:**
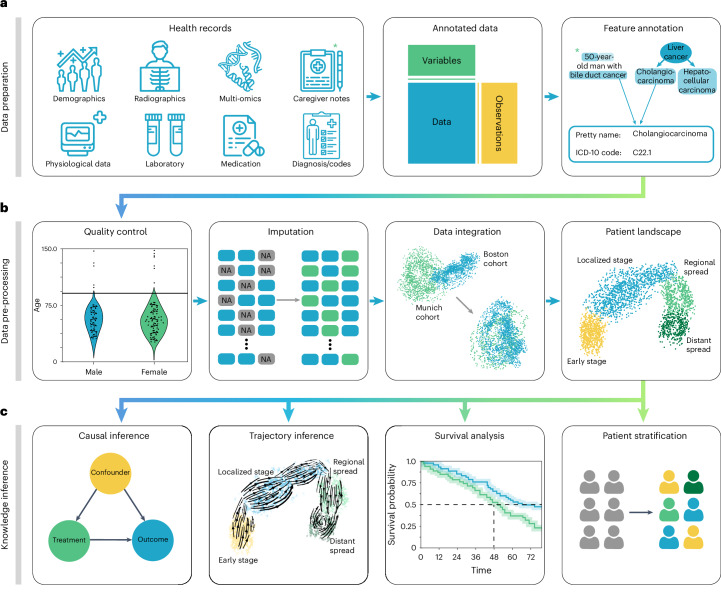
Schematic overview of EHR analysis with ehrapy. **a**, Heterogeneous health data are first loaded into memory as an AnnData object with patient visits as observational rows and variables as columns. Next, the data can be mapped against ontologies, and key terms are extracted from free text notes. **b**, The EHR data are subject to quality control where low-quality or spurious measurements are removed or imputed. Subsequently, numerical data are normalized, and categorical data are encoded. Data from different sources with data distribution shifts are integrated, embedded, clustered and annotated in a patient landscape. **c**, Further downstream analyses depend on the question of interest and can include the inference of causal effects and trajectories, survival analysis or patient stratification.

Powered by scanpy, which scales to millions of observations^52^ (Methods and Supplementary Table 1) and the machine learning library scikit-learn^53^, ehrapy provides more than 100 composable analysis functions organized in modules from which custom analysis pipelines can be built. Each function directly interacts with the AnnData object and adds all intermediate results for simple access and reuse of information to it. To facilitate setting up these pipelines, ehrapy guides analysts through a general analysis pipeline (Fig. 1). At any step of an analysis pipeline, community software packages can be integrated without any vendor lock-in. Because ehrapy is built on open standards, it can be purposefully extended to solve new challenges, such as the development of foundational models (Methods).

In the ehrapy analysis pipeline, EHR data are initially inspected for quality issues by analyzing feature distributions that may skew results and by detecting visits and features with high missing rates that ehrapy can then impute (Methods). ehrapy tracks all filtering steps while keeping track of population dynamics to highlight potential selection and filtering biases (Methods). Subsequently, ehrapy’s normalization and encoding functions (Methods) are applied to achieve a uniform numerical representation that facilitates data integration and corrects for dataset shift effects (Methods). Calculated lower-dimensional representations can subsequently be visualized, clustered and annotated to obtain a patient landscape (Methods). Such annotated groups of patients can be used for statistical comparisons to find differences in features among them to ultimately learn markers of patient states.

As analysis goals can differ between users and datasets, the ehrapy analysis pipeline is customizable during the final knowledge inference step. ehrapy provides statistical methods for group comparison and extensive support for survival analysis (Methods), enabling the discovery of biomarkers. Furthermore, ehrapy offers functions for causal inference to go from statistically determined associations to causal relations (Methods). Moreover, patient visits in aggregated EHR data can be regarded as snapshots where individual measurements taken at specific timepoints might not adequately reflect the underlying progression of disease and result from unrelated variation due to, for example, day-to-day differences^54–56^. Therefore, disease progression models should rely on analysis of the underlying clinical data, as disease progression in an individual patient may not be monotonous in time. ehrapy allows for the use of advanced trajectory inference methods to overcome sparse measurements^57–59^. We show that this approach can order snapshots to calculate a pseudotime that can adequately reflect the progression of the underlying clinical process. Given a sufficient number of snapshots, ehrapy increases the potential to understand disease progression, which is likely not robustly captured within a single EHR but, rather, across several.

### ehrapy enables patient stratification in pneumonia cases

**Fig. 2:**
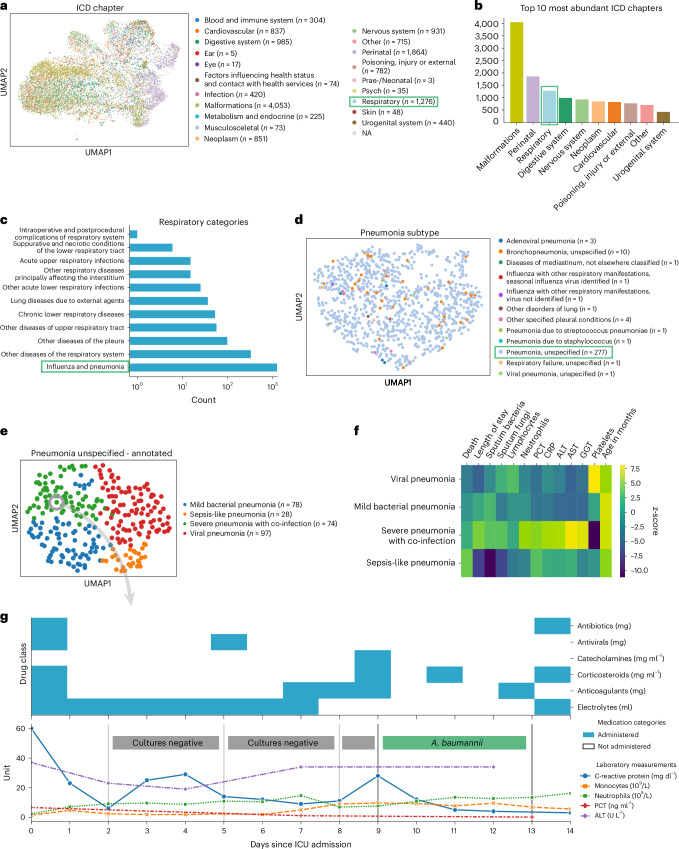
PIC dataset overview and annotation of patients diagnosed with unspecified pneumonia. **a**, UMAP of all patient visits in the ICU with primary discharge diagnosis grouped by ICD chapter. **b**, The prevalence of respiratory diseases prompted us to investigate them further. **c**, Respiratory categories show the abundance of influenza and pneumonia diagnoses that we investigated more closely. **d**, We observed the ‘unspecified pneumonia’ subgroup, which led us to investigate and annotate it in more detail. **e**, The previously ‘unspecified pneumonia’-labeled patients were annotated using several clinical features (Extended Data Fig. 5), of which the most important ones are shown in the heatmap (**f**). **g**, Example disease progression of an individual child with pneumonia illustrating pharmacotherapy over time until positive *A. baumannii* swab.

**Extended Data Fig. 1:**
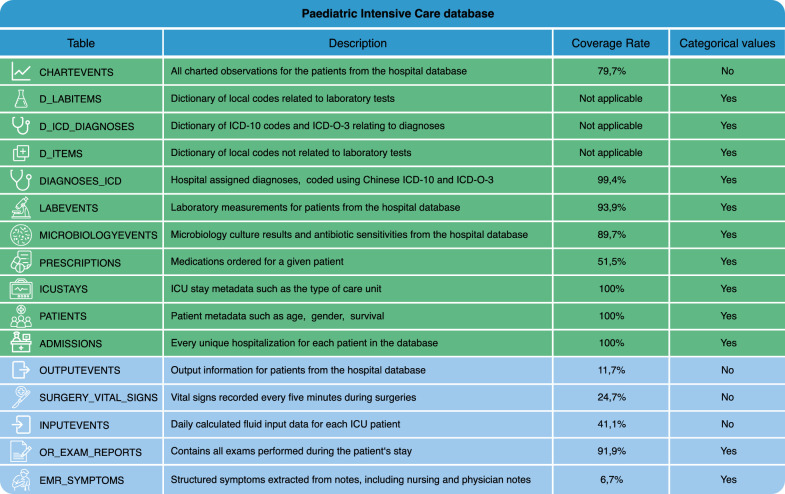
Overview of the paediatric intensive care database (PIC). The database consists of several tables corresponding to several data modalities and measurement types. All tables colored in green were selected for analysis and all tables in blue were discarded based on coverage rate. Despite the high coverage rate, we discarded the ‘OR_EXAM_REPORTS’ table because of the lack of detail in the exam reports.

**Extended Data Fig. 2:**
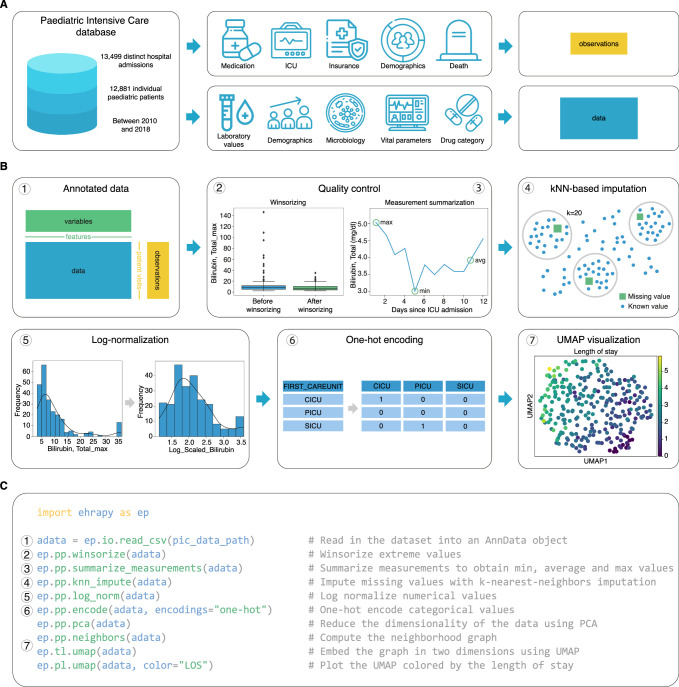
Preprocessing of the Paediatric Intensive Care (PIC) dataset with ehrapy. (**a**) Heterogeneous data of the PIC database was stored in ‘data’ (matrix that is used for computations) and ‘observations’ (metadata per patient visit). During quality control, further annotations are added to the ‘variables’ (metadata per feature) slot. (**b**) Preprocessing steps of the PIC dataset. (**c**) Example of the function calls in the data analysis pipeline that resembles the preprocessing steps in (B) using ehrapy.

**Extended Data Fig. 3:**
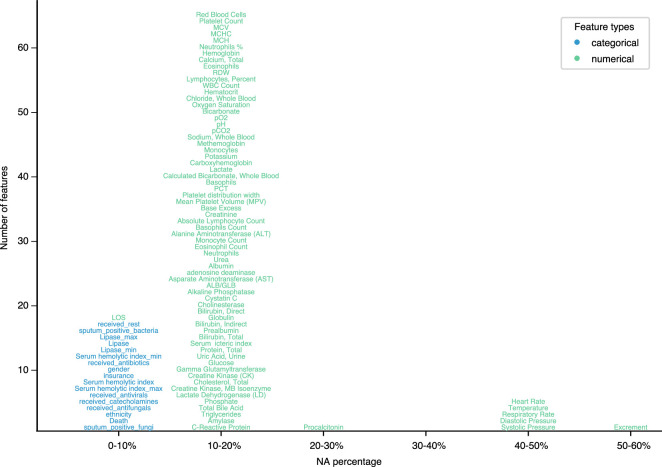
Missing data distribution for the ‘youths’ group of the PIC dataset. The x-axis represents the percentage of missing values in each feature. The y-axis reflects the number of features in each bin with text labels representing the names of the individual features.

To demonstrate ehrapy’s capability to analyze heterogeneous datasets from a broad patient set across multiple care units, we applied our exploratory strategy to the PIC^43^ database. The PIC database is a single-center database hosting information on children admitted to critical care units at the Children’s Hospital of Zhejiang University School of Medicine in China. It contains 13,499 distinct hospital admissions of 12,881 individual pediatric patients admitted between 2010 and 2018 for whom demographics, diagnoses, doctors’ notes, vital signs, laboratory and microbiology tests, medications, fluid balances and more were collected (Extended Data Figs. 1 and 2a and Methods). After missing data imputation and subsequent pre-processing (Extended Data Figs. 2b,c and 3 and Methods), we generated a uniform manifold approximation and projection (UMAP) embedding to visualize variation across all patients using ehrapy (Fig. 2a). This visualization of the low-dimensional patient manifold shows the heterogeneity of the collected data in the PIC database, with malformations, perinatal and respiratory being the most abundant International Classification of Diseases (ICD) chapters (Fig. 2b). The most common respiratory disease categories (Fig. 2c) were labeled pneumonia and influenza (*n* = 984). We focused on pneumonia to apply ehrapy to a challenging, broad-spectrum disease that affects all age groups. Pneumonia is a prevalent respiratory infection that poses a substantial burden on public health^60^ and is characterized by inflammation of the alveoli and distal airways^60^. Individuals with pre-existing chronic conditions are particularly vulnerable, as are children under the age of 5 (ref. ^61^). Pneumonia can be caused by a range of microorganisms, encompassing bacteria, respiratory viruses and fungi.

**Extended Data Fig. 4:**
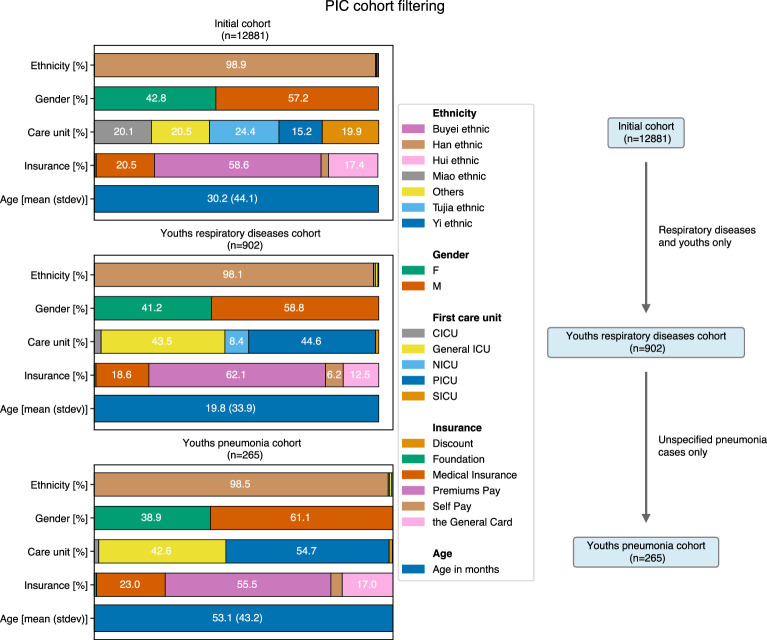
Patient selection during analysis of the PIC dataset. Filtering for the pneumonia cohort of the youths filters out care units except for the general intensive care unit and the pediatric intensive care unit.

We selected the age group ‘youths’ (13 months to 18 years of age) for further analysis, addressing a total of 265 patients who dominated the pneumonia cases and were diagnosed with ‘unspecified pneumonia’ (Fig. 2d and Extended Data Fig. 4). Neonates (0–28 d old) and infants (29 d to 12 months old) were excluded from the analysis as the disease context is significantly different in these age groups due to distinct anatomical and physical conditions. Patients were 61% male, had a total of 277 admissions, had a mean age at admission of 54 months (median, 38 months) and had an average LOS of 15 d (median, 7 d). Of these, 152 patients were admitted to the pediatric intensive care unit (PICU), 118 to the general ICU (GICU), four to the surgical ICU (SICU) and three to the cardiac ICU (CICU). Laboratory measurements typically had 12–14% missing data, except for serum procalcitonin (PCT), a marker for bacterial infections, with 24.5% missing, and C-reactive protein (CRP), a marker of inflammation, with 16.8% missing. Measurements assigned as ‘vital signs’ contained between 44% and 54% missing values. Stratifying patients with unspecified pneumonia further enables a more nuanced understanding of the disease, potentially facilitating tailored approaches to treatment.

**Extended Data Fig. 5:**
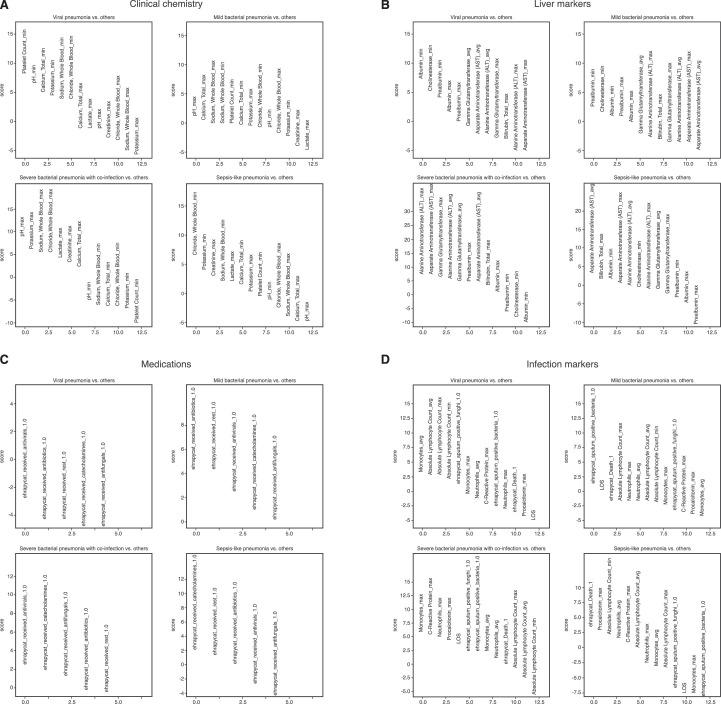
Feature rankings of stratified patient groups. Scores reflect the z-score underlying the p-value per measurement for each group. Higher scores (above 0) reflect overrepresentation of the measurement compared to all other groups and vice versa. (**a**) By clinical chemistry. (**b**) By liver markers. (**c**) By medication type. (**d**) By infection markers.

To deepen clinical phenotyping for the disease group ‘unspecified pneumonia’, we calculated a *k*-nearest neighbor graph to cluster patients into groups and visualize these in UMAP space (Methods). Leiden clustering^62^ identified four patient groupings with distinct clinical features that we annotated (Fig. 2e). To identify the laboratory values, medications and pathogens that were most characteristic for these four groups (Fig. 2f), we applied *t*-tests for numerical data and *g*-tests for categorical data between the identified groups using ehrapy (Extended Data Fig. 5 and Methods). Based on this analysis, we identified patient groups with ‘sepsis-like, ‘severe pneumonia with co-infection’, ‘viral pneumonia’ and ‘mild pneumonia’ phenotypes. The ‘sepsis-like’ group of patients (*n* = 28) was characterized by rapid disease progression as exemplified by an increased number of deaths (adjusted *P* ≤ 5.04 × 10^−3^, 43% (*n* = 28), 95% confidence interval (CI): 23%, 62%); indication of multiple organ failure, such as elevated creatinine (adjusted *P* ≤ 0.01, 52.74 ± 23.71 μmol L^−1^) or reduced albumin levels (adjusted *P* ≤ 2.89 × 10^−4^, 33.40 ± 6.78 g L^−1^); and increased expression levels and peaks of inflammation markers, including PCT (adjusted *P* ≤ 3.01 × 10^−2^, 1.42 ± 2.03 ng ml^−1^), whole blood cell count, neutrophils, lymphocytes, monocytes and lower platelet counts (adjusted *P* ≤ 6.3 × 10^−2^, 159.30 ± 142.00 × 10^9^ per liter) and changes in electrolyte levels—that is, lower potassium levels (adjusted *P* ≤ 0.09 × 10^−2^, 3.14 ± 0.54 mmol L^−1^). Patients whom we associated with the term ‘severe pneumonia with co-infection’ (*n* = 74) were characterized by prolonged ICU stays (adjusted *P* ≤ 3.59 × 10^−4^, 15.01 ± 29.24 d); organ affection, such as higher levels of creatinine (adjusted *P* ≤ 1.10 × 10^−4^, 52.74 ± 23.71 μmol L^−1^) and lower platelet count (adjusted *P* ≤ 5.40 × 10^−23^, 159.30 ± 142.00 × 10^9^ per liter); increased inflammation markers, such as peaks of PCT (adjusted *P* ≤ 5.06 × 10^−5^, 1.42 ± 2.03 ng ml^−1^), CRP (adjusted *P* ≤ 1.40 × 10^−6^, 50.60 ± 37.58 mg L^−1^) and neutrophils (adjusted *P* ≤ 8.51 × 10^−6^, 13.01 ± 6.98 × 10^9^ per liter); detection of bacteria in combination with additional pathogen fungals in sputum samples (adjusted *P* ≤ 1.67 × 10^−2^, 26% (*n* = 74), 95% CI: 16%, 36%); and increased application of medication, including antifungals (adjusted *P* ≤ 1.30 × 10^−4^, 15% (*n* = 74), 95% CI: 7%, 23%) and catecholamines (adjusted *P* ≤ 2.0 × 10^−2^, 45% (*n* = 74), 95% CI: 33%, 56%). Patients in the ‘mild pneumonia’ group were characterized by positive sputum cultures in the presence of relatively lower inflammation markers, such as PCT (adjusted *P* ≤ 1.63 × 10^−3^, 1.42 ± 2.03 ng ml^−1^) and CRP (adjusted *P* ≤ 0.03 × 10^−1^, 50.60 ± 37.58 mg L^−1^), while receiving antibiotics more frequently (adjusted *P* ≤ 1.00 × 10^−5^, 80% (*n* = 78), 95% CI: 70%, 89%) and additional medications (electrolytes, blood thinners and circulation-supporting medications) (adjusted *P* ≤ 1.00 × 10^−5^, 82% (*n* = 78), 95% CI: 73%, 91%). Finally, patients in the ‘viral pneumonia’ group were characterized by shorter LOSs (adjusted *P* ≤ 8.00 × 10^−6^, 15.01 ± 29.24 d), a lack of non-viral pathogen detection in combination with higher lymphocyte counts (adjusted *P* ≤ 0.01, 4.11 ± 2.49 × 10^9^ per liter), lower levels of PCT (adjusted *P* ≤ 0.03 × 10^−2^, 1.42 ± 2.03 ng ml^−1^) and reduced application of catecholamines (adjusted *P* ≤ 5.96 × 10^−7^, 15% (n = 97), 95% CI: 8%, 23%), antibiotics (adjusted *P* ≤ 8.53 × 10^−6^, 41% (*n* = 97), 95% CI: 31%, 51%) and antifungals (adjusted *P* ≤ 5.96 × 10^−7^, 0% (*n* = 97), 95% CI: 0%, 0%).

To demonstrate the ability of ehrapy to examine EHR data from different levels of resolution, we additionally reconstructed a case from the ‘severe pneumonia with co-infection’ group (Fig. 2g). In this case, the analysis revealed that CRP levels remained elevated despite broad-spectrum antibiotic treatment until a positive *Acinetobacter baumannii* result led to a change in medication and a subsequent decrease in CRP and monocyte levels.

### ehrapy facilitates extraction of pneumonia indicators

**Fig. 3:**
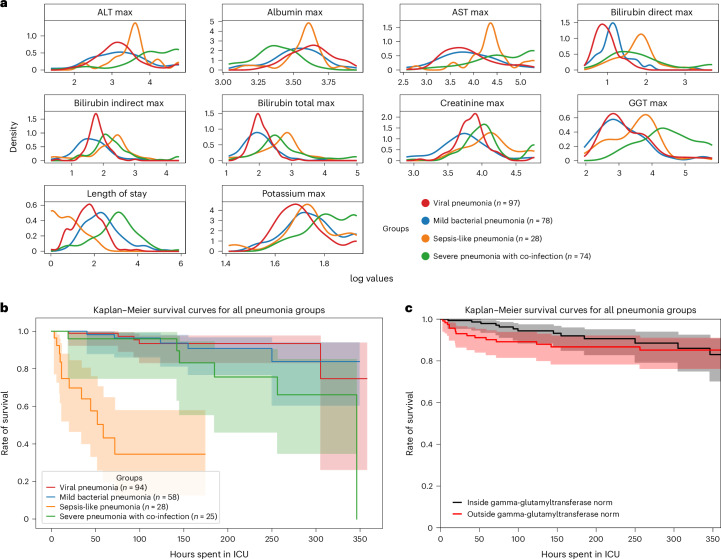
Survival analysis of patients diagnosed with unspecified pneumonia. **a**, Line plots of major hepatic system laboratory measurements per group show variance in the measurements per pneumonia group. **b**, Kaplan–Meier survival curves demonstrate lower survival for ‘sepsis-like’ and ‘severe pneumonia with co-infection’ groups. **c**, Kaplan–Meier survival curves for children with GGT measurements outside the norm range display lower survival.

**Extended Data Fig. 6:**
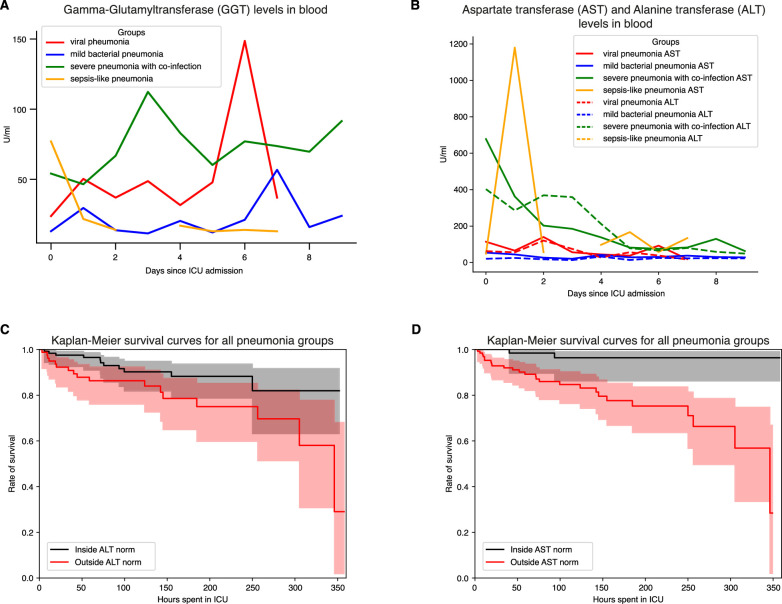
Liver marker value progression for the ‘youths’ group and Kaplan-Meier curves. (**a**) Viral and severe pneumonia with co-infection groups display enriched gamma-glutamyl transferase levels in blood serum. (**b**) Aspartate transferase (AST) and Alanine transaminase (ALT) levels are enriched for severe pneumonia with co-infection during early ICU stay. (**c**) and (**d**) Kaplan-Meier curves for ALT and AST demonstrate lower survivability for children with measurements outside the norm.

ehrapy’s survival analysis module allowed us to identify clinical indicators of disease stages that could be used as biomarkers through Kaplan–Meier analysis. We found strong variance in overall aspartate aminotransferase (AST), alanine aminotransferase (ALT), gamma-glutamyl transferase (GGT) and bilirubin levels (Fig. 3a), including changes over time (Extended Data Fig. 6a,b), in all four ‘unspecified pneumonia’ groups. Routinely used to assess liver function, studies provide evidence that AST, ALT and GGT levels are elevated during respiratory infections^63^, including severe pneumonia^64^, and can guide diagnosis and management of pneumonia in children^63^. We confirmed reduced survival in more severely affected children (‘sepsis-like pneumonia’ and ‘severe pneumonia with co-infection’) using Kaplan–Meier curves and a multivariate log-rank test (Fig. 3b; *P* ≤ 1.09 × 10^−18^) through ehrapy. To verify the association of this trajectory with altered AST, ALT and GGT expression levels, we further grouped all patients based on liver enzyme reference ranges (Methods and Supplementary Table 2). By Kaplan–Meier survival analysis, cases with peaks of GGT (*P* ≤ 1.4 × 10^−2^, 58.01 ± 2.03 U L^−1^), ALT (*P* ≤ 2.9 × 10^−2^, 43.59 ± 38.02 U L^−1^) and AST (*P* ≤ 4.8 × 10^−4^, 78.69 ± 60.03 U L^−1^) in ‘outside the norm’ were found to correlate with lower survival in all groups (Fig. 3c and Extended Data Fig. 6), in line with previous studies^63,65^. Bilirubin was not found to significantly affect survival (*P* ≤ 2.1 × 10^−1^, 12.57 ± 21.22 mg dl^−1^).

### ehrapy quantifies medication class effect on LOS

**Fig. 4:**
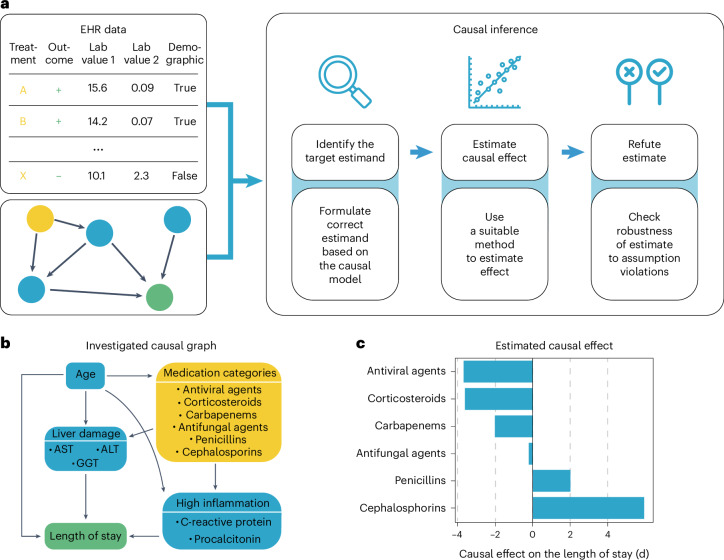
Causal inference of LOS affected by different medication types. **a**, ehrapy’s causal module is based on the strategy of the tool ‘dowhy’. Here, EHR data containing treatment, outcome and measurements and a causal graph serve as input for causal effect quantification. The process includes the identification of the target estimand based on the causal graph, the estimation of causal effects using various models and, finally, refutation where sensitivity analyses and refutation tests are performed to assess the robustness of the results and assumptions. **b**, Curated causal graph using age, liver damage and inflammation markers as disease progression proxies together with medications as interventions to assess the causal effect on length of ICU stay. **c**, Determined causal effect strength on LOS in days of administered medication categories.

Pneumonia requires case-specific medications due to its diverse causes. To demonstrate the potential of ehrapy’s causal inference module, we quantified the effect of medication on ICU LOS to evaluate case-specific administration of medication. In contrast to causal discovery that attempts to find a causal graph reflecting the causal relationships, causal inference is a statistical process used to investigate possible effects when altering a provided system, as represented by a causal graph and observational data (Fig. 4a)^66^. This approach allows identifying and quantifying the impact of specific interventions or treatments on outcome measures, thereby providing insight for evidence-based decision-making in healthcare. Causal inference relies on datasets incorporating interventions to accurately quantify effects.

**Extended Data Fig. 7:**
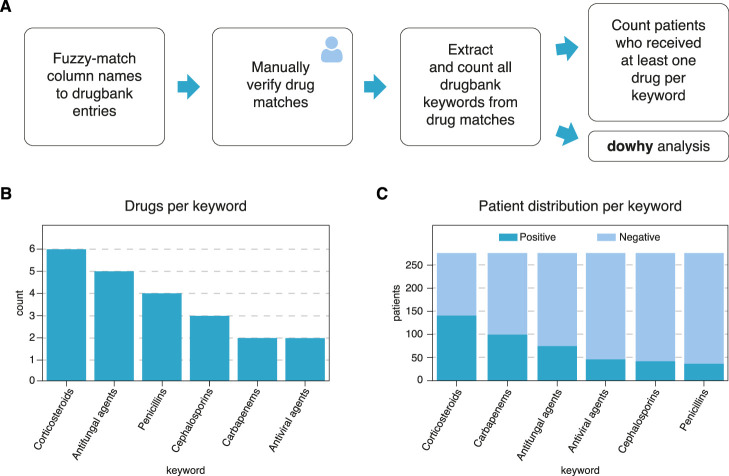
Overview of medication categories used for causal inference. (**a**) Feature engineering process to group administered medications into medication categories using drugbank. (**b**) Number of medications per medication category. (**c**) Number of patients that received (dark blue) and did not receive specific medication categories (light blue).

We manually constructed a minimal causal graph with ehrapy (Fig. 4b) on records of treatment with corticosteroids, carbapenems, penicillins, cephalosporins and antifungal and antiviral medications as interventions (Extended Data Fig. 7 and Methods). We assumed that the medications affect disease progression proxies, such as inflammation markers and markers of organ function. The selection of ‘interventions’ is consistent with current treatment standards for bacterial pneumonia and respiratory distress^67,68^. Based on the approach of the tool ‘dowhy’^69^ (Fig. 4a), ehrapy’s causal module identified the application of corticosteroids, antivirals and carbapenems to be associated with shorter LOSs, in line with current evidence^61,70–72^. In contrast, penicillins and cephalosporins were associated with longer LOSs, whereas antifungal medication did not strongly influence LOS (Fig. 4c).

### ehrapy enables deriving population-scale risk factors

**Fig. 5:**
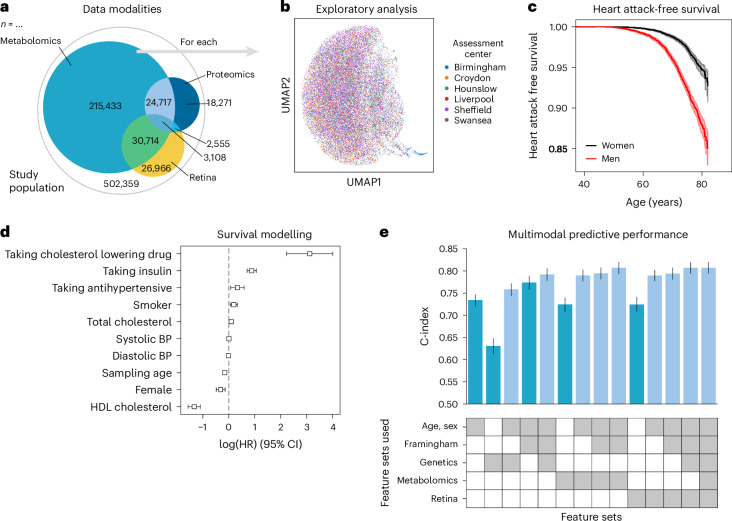
Analysis of myocardial infarction risk in the UKB. **a**, The UKB includes 502,359 participants from 22 assessment centers. Most participants have genetic data (97%) and physical measurement data (93%), but fewer have data for complex measures, such as metabolomics, retinal imaging or proteomics. **b**, We found a distinct cluster of individuals (bottom right) from the Birmingham assessment center in the retinal imaging data, which is an artifact of the image acquisition process and was, thus, excluded. **c**, Myocardial infarctions are recorded for 15% of the male and 7% of the female study population. Kaplan–Meier estimators with 95% CIs are shown. **d**, For every modality combination, a linear Cox proportional hazards model was fit to determine the prognostic potential of these for myocardial infarction. Cardiovascular risk factors show expected positive log hazard ratios (log (HRs)) for increased blood pressure or total cholesterol and negative ones for sampling age and systolic blood pressure (BP). log (HRs) with 95% CIs are shown. **e**, Combining all features yields a C-index of 0.81. **c**–**e**, Error bars indicate 95% CIs (*n* = 29,216).

**Extended Data Fig. 8:**
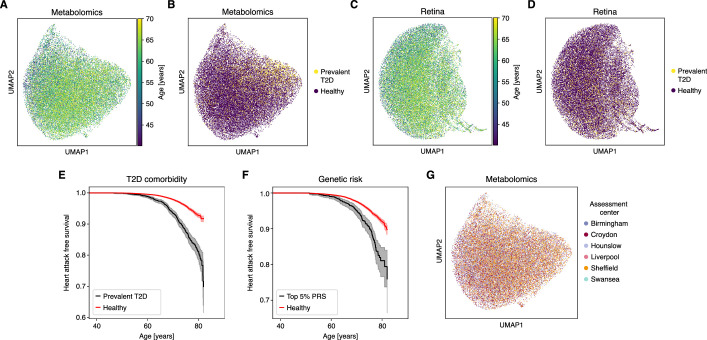
UK-Biobank data overview and quality control across modalities. (**a**) UMAP plot of the metabolomics data demonstrating a clear gradient with respect to age at sampling, and (**b**) type 2 diabetes prevalence. (**c**) Analogously, the features derived from retinal imaging show a less pronounced age gradient, and (**d**) type 2 diabetes prevalence gradient. (**e**) Stratifying myocardial infarction risk by the type 2 diabetes comorbidity confirms vastly increased risk with a prior type 2 (T2D) diabetes diagnosis. Kaplan-Meier estimators with 95 % confidence intervals are shown. (**f**) Similarly, the polygenic risk score for coronary heart disease used in this work substantially enriches myocardial infarction risk in its top 5% percentile. Kaplan-Meier estimators with 95 % confidence intervals are shown. (**g**) UMAP visualization of the metabolomics features colored by the assessment center shows no discernable biases. (A-G) n = 29,216.

**Extended Data Fig. 9:**
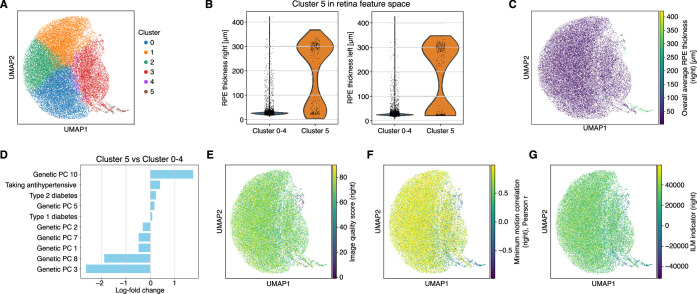
UK-Biobank retina derived feature quality control. (**a**) Leiden Clustering of retina derived feature space. (**b**) Comparison of ‘overall retinal pigment epithelium (RPE) thickness’ values between cluster 5 (n = 301) and the rest of the population (n = 28,915). (**c**) RPE thickness in the right eye outliers on the UMAP largely corresponds to cluster 5. (**d**) Log ratio of top and bottom 5 fields in obs dataframe between cluster 5 and the rest of the population. (**e**) Image Quality of the optical coherence tomography scan as reported in the UKB. (**f**) Minimum motion correlation quality control indicator. (**g**) Inner limiting membrane (ILM) quality control indicator. (D-G) Data are shown for the right eye only, comparable results for the left eye are omitted. (A-G) n = 29,216.

To illustrate the advantages of using a unified data management and quality control framework, such as ehrapy, we modeled myocardial infarction risk using Cox proportional hazards models on UKB^44^ data. Large population cohort studies, such as the UKB, enable the investigation of common diseases across a wide range of modalities, including genomics, metabolomics, proteomics, imaging data and common clinical variables (Fig. 5a,b). From these, we used a publicly available polygenic risk score for coronary heart disease^73^ comprising 6.6 million variants, 80 nuclear magnetic resonance (NMR) spectroscopy-based metabolomics^74^ features, 81 features derived from retinal optical coherence tomography^75,76^ and the Framingham Risk Score^77^ feature set, which includes known clinical predictors, such as age, sex, body mass index, blood pressure, smoking behavior and cholesterol levels. We excluded features with more than 10% missingness and imputed the remaining missing values (Methods). Furthermore, individuals with events up to 1 year after the sampling time were excluded from the analyses, ultimately selecting 29,216 individuals for whom all mentioned data types were available (Extended Data Figs. 8 and 9 and Methods). Myocardial infarction, as defined by our mapping to the phecode nomenclature^51^, was defined as the endpoint (Fig. 5c). We modeled the risk for myocardial infarction 1 year after either the metabolomic sample was obtained or imaging was performed.

Predictive performance for each modality was assessed by fitting Cox proportional hazards (Fig. 5c) models on each of the feature sets using ehrapy (Fig. 5d). The age of the first occurrence served as the time to event; alternatively, date of death or date of the last record in the EHR served as censoring times. Models were evaluated using the concordance index (C-index) (Methods). The combination of multiple modalities successfully improved the predictive performance for coronary heart disease by increasing the C-index from 0.63 (genetic) to 0.76 (genetics, age and sex) and to 0.77 (clinical predictors) with 0.81 (imaging and clinical predictors) for combinations of feature sets (Fig. 5e). Our finding is in line with previous observations of complementary effects between different modalities, where a broader ‘major adverse cardiac event’ phenotype was modeled in the UKB achieving a C-index of 0.72 (ref. ^78^). Adding genetic data improves predictive potential, as it is independent of sampling age and has limited prediction of other modalities^79^. The addition of metabolomic data did not improve predictive power (Fig. 5e).

### Imaging-based disease severity projection via fate mapping

To demonstrate ehrapy’s ability to handle diverse image data and recover disease stages, we embedded pulmonary imaging data obtained from patients with COVID-19 into a lower-dimensional space and computationally inferred disease progression trajectories using pseudotemporal ordering. This describes a continuous trajectory or ordering of individual points based on feature similarity^80^. Continuous trajectories enable mapping the fate of new patients onto precise states to potentially predict their future condition.

In COVID-19, a highly contagious respiratory illness caused by severe acute respiratory syndrome coronavirus 2 (SARS-CoV-2), symptoms range from mild flu-like symptoms to severe respiratory distress. Chest x-rays typically show opacities (bilateral patchy, ground glass) associated with disease severity^81^.

**Fig. 6:**
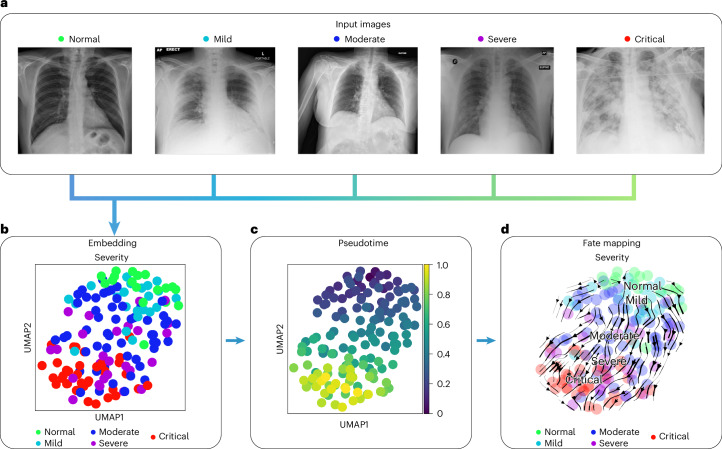
Recovery of disease severity trajectory in COVID-19 chest x-ray images. **a**, Randomly selected chest x-ray images from the BrixIA dataset demonstrate its variance. **b**, UMAP visualization of the BrixIA dataset embedding shows a separation of disease severity classes. **c**, Calculated pseudotime for all images increases with distance to the ‘normal’ images. **d**, Stream projection of fate mapping in UMAP space showcases disease severity trajectory of the COVID-19 chest x-ray images.

We used COVID-19 chest x-ray images from the BrixIA^82^ dataset consisting of 192 images (Fig. 6a) with expert annotations of disease severity. We used the BrixIA database scores, which are based on six regions annotated by radiologists, to classify disease severity (Methods). We embedded raw image features using a pre-trained DenseNet model (Methods) and further processed this embedding into a nearest-neighbors-based UMAP space using ehrapy (Fig. 6b and Methods). Fate mapping based on imaging information (Methods) determined a severity ordering from mild to critical cases (Fig. 6b–d). Images labeled as ‘normal’ are projected to stay within the healthy group, illustrating the robustness of our approach. Images of diseased patients were ordered by disease severity, highlighting clear trajectories from ‘normal’ to ‘critical’ states despite the heterogeneity of the x-ray images stemming from, for example, different zoom levels (Fig. 6a).

### Detecting and mitigating biases in EHR data with ehrapy

To showcase how exploratory analysis using ehrapy can reveal and mitigate biases, we analyzed the Fairlearn^83^ version of the Diabetes 130-US Hospitals^84^ dataset. The dataset covers 10 years (1999–2008) of clinical records from 130 US hospitals, detailing 47 features of diabetes diagnoses, laboratory tests, medications and additional data from up to 14 d of inpatient care of 101,766 diagnosed patient visits (Methods). It was originally collected to explore the link between the measurement of hemoglobin A1c (HbA1c) and early readmission.

**Extended Data Fig. 10:**
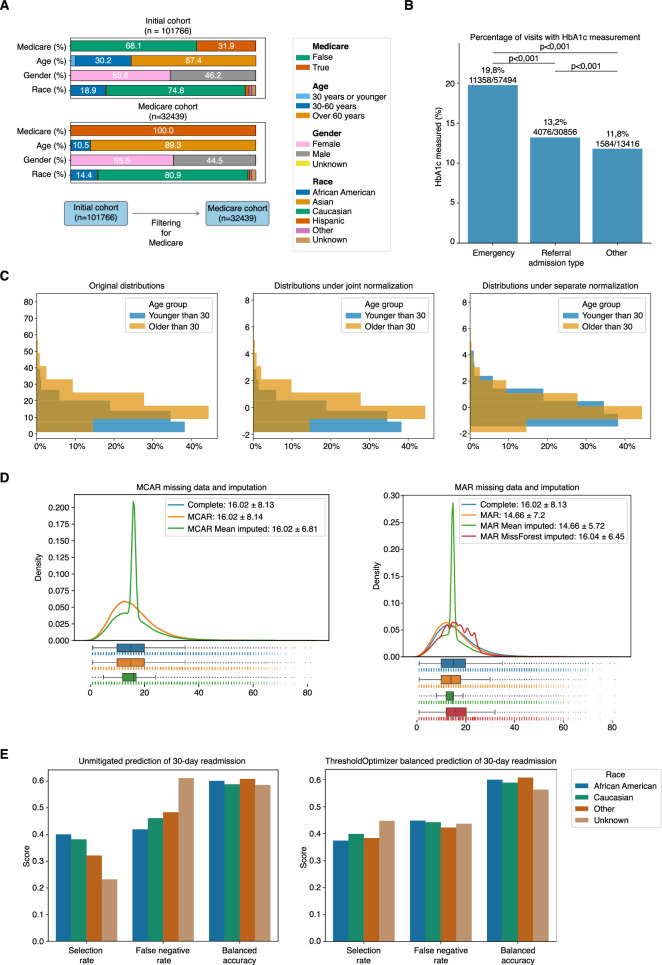
Bias detection and mitigation study on the Diabetes 130-US hospitals dataset (n = 101,766 hospital visits, one patient can have multiple visits). (**a**) Filtering to the visits of Medicare recipients results in an increase of Caucasians. (**b**) Proportion of visits where Hb1Ac measurements are recorded, stratified by admission type. Adjusted P values were calculated with Chi squared tests and Bonferroni correction (Adjusted P values: Emergency vs Referral 3.3E-131, Emergency vs Other 1.4E-101, Referral vs Other 1.6E-4.) (**c**) Normalizing feature distributions jointly vs. separately can mask distribution differences. (**d**) Imputing the number of medications for visits. Onto the complete data (blue), MCAR (30% missing data) and MAR (38% missing data) were introduced (orange), with the MAR mechanism depending on the time in hospital. Mean imputation (green) can reduce the variance of the distribution under MCAR and MAR mechanisms, and bias the center of the distribution under an MAR mechanism. Multiple imputation, such as MissForest imputation can impute meaningfully even in MAR cases, when having access to variables involved in the MAR mechanism. Each boxplot represents the IQR of the data, with the horizontal line inside the box indicating the median value. The left and right bounds of the box represent the first and third quartiles, respectively. The ‘whiskers’ extend to the minimum and maximum values within 1.5 times the IQR from the lower and upper quartiles, respectively. (**e**) Predicting the early readmission within 30 days after release on a per-stay level. Balanced accuracy can mask differences in selection and false negative rate between sensitive groups.

The cohort primarily consists of White and African American individuals, with only a minority of cases from Asian or Hispanic backgrounds (Extended Data Fig. 10a). ehrapy’s cohort tracker unveiled selection and surveillance biases when filtering for Medicare recipients for further analysis, resulting in a shift of age distribution toward an age of over 60 years in addition to an increasing ratio of White participants. Using ehrapy’s visualization modules, our analysis showed that HbA1c was measured in only 18.4% of inpatients, with a higher frequency in emergency admissions compared to referral cases (Extended Data Fig. 10b). Normalization biases can skew data relationships when standardization techniques ignore subgroup variability or assume incorrect distributions. The choice of normalization strategy must be carefully considered to avoid obscuring important factors. When normalizing the number of applied medications individually, differences in distributions between age groups remained. However, when normalizing both distributions jointly with age group as an additional group variable, differences between age groups were masked (Extended Data Fig. 10c). To investigate missing data and imputation biases, we introduced missingness for the number of applied medications according to an MCAR mechanism, which we verified using ehrapy’s Little’s test (*P* ≤ 0.01 × 10^−2^), and an MAR mechanism (Methods). Whereas imputing the mean in the MCAR case did not affect the overall location of the distribution, it led to an underestimation of the variance, with the standard deviation dropping from 8.1 in the original data to 6.8 in the imputed data (Extended Data Fig. 10d). Mean imputation in the MAR case skewed both location and variance of the mean from 16.02 to 14.66, with a standard deviation of only 5.72 (Extended Data Fig. 10d). Using ehrapy’s multiple imputation based MissForest^85^ imputation on the MAR data resulted in a mean of 16.04 and a standard deviation of 6.45. To predict patient readmission in fewer than 30 d, we merged the three smallest race groups, ‘Asian’, ‘Hispanic’ and ‘Other’. Furthermore, we dropped the gender group ‘Unknown/Invalid’ owing to the small sample size making meaningful assessment impossible, and we performed balanced random undersampling, resulting in 5,677 cases from each condition. We observed an overall balanced accuracy of 0.59 using a logistic regression model. However, the false-negative rate was highest for the races ‘Other’ and ‘Unknown’, whereas their selection rate was lowest, and this model was, therefore, biased (Extended Data Fig. 10e). Using ehrapy’s compatibility with existing machine learning packages, we used Fairlearn’s ThresholdOptimizer (Methods), which improved the selection rates for ‘Other’ from 0.32 to 0.38 and for ‘Unknown’ from 0.23 to 0.42 and the false-negative rates for ‘Other’ from 0.48 to 0.42 and for ‘Unknown’ from 0.61 to 0.45 (Extended Data Fig. 10e).

## Discussion

Clustering offers a hypothesis-free alternative to supervised classification when clear hypotheses or labels are missing. It has enabled the identification of heart failure subtypes^86^ and progression pathways^87^ and COVID-19 severity states^88^. This concept, which is central to ehrapy, further allowed us to identify fine-grained groups of ‘unspecified pneumonia’ cases in the PIC dataset while discovering biomarkers and quantifying effects of medications on LOS. Such retroactive characterization showcases ehrapy’s ability to put complex evidence into context. This approach supports feedback loops to improve diagnostic and therapeutic strategies, leading to more efficiently allocated resources in healthcare.

ehrapy’s flexible data structures enabled us to integrate the heterogeneous UKB data for predictive performance in myocardial infarction. The different data types and distributions posed a challenge for predictive models that were overcome with ehrapy’s pre-processing modules. Our analysis underscores the potential of combining phenotypic and health data at population scale through ehrapy to enhance risk prediction.

By adapting pseudotime approaches that are commonly used in other omics domains, we successfully recovered disease trajectories from raw imaging data with ehrapy. The determined pseudotime, however, only orders data but does not necessarily provide a future projection per patient. Understanding the driver features for fate mapping in image-based datasets is challenging. The incorporation of image segmentation approaches could mitigate this issue and provide a deeper insight into the spatial and temporal dynamics of disease-related processes.

Limitations of our analyses include the lack of control for informative missingness where the absence of information represents information in itself^89^. Translation from Chinese to English in the PIC database can cause information loss and inaccuracies because the Chinese ICD-10 codes are seven characters long compared to the five-character English codes. Incompleteness of databases, such as the lack of radiology images in the PIC database, low sample sizes, underrepresentation of non-White ancestries and participant self-selection, cannot be accounted for and limit generalizability. This restricts deeper phenotyping of, for example, all ‘unspecified pneumonia’ cases with respect to their survival, which could be overcome by the use of multiple databases. Our causal inference use case is limited by unrecorded variables, such as Sequential Organ Failure Assessment (SOFA) scores, and pneumonia-related pathogens that are missing in the causal graph due to dataset constraints, such as high sparsity and substantial missing data, which risk overfitting and can lead to overinterpretation. We counterbalanced this by employing several refutation methods that statistically reject the causal hypothesis, such as a placebo treatment, a random common cause or an unobserved common cause. The longer hospital stays associated with penicillins and cephalosporins may be dataset specific and stem from higher antibiotic resistance, their use as first-line treatments, more severe initial cases, comorbidities and hospital-specific protocols.

Most analysis steps can introduce algorithmic biases where results are misleading or unfavorably affect specific groups. This is particularly relevant in the context of missing data^22^ where determining the type of missing data is necessary to handle it correctly. ehrapy includes an implementation of Little’s test^90^, which tests whether data are distributed MCAR to discern missing data types. For MCAR data single-imputation approaches, such as mean, median or mode, imputation can suffice, but these methods are known to reduce variability^91,92^. Multiple imputation strategies, such as Multiple Imputation by Chained Equations (MICE)^93^ and MissForest^85^, as implemented in ehrapy, are effective for both MCAR and MAR data^22,94,95^. MNAR data require pattern-mixture or shared-parameter models that explicitly incorporate the mechanism by which data are missing^96^. Because MNAR involves unobserved data, the assumptions about the missingness mechanism cannot be directly verified, making sensitivity analysis crucial^21^. ehrapy’s wide range of normalization functions and grouping functionality enables to account for intrinsic variability within subgroups, and its compatibility with Fairlearn^83^ can potentially mitigate predictor biases. Generally, we recommend to assess all pre-processing in an iterative manner with respect to downstream applications, such as patient stratification. Moreover, sensitivity analysis can help verify the robustness of all inferred knowledge^97^.

These diverse use cases illustrate ehrapy’s potential to sufficiently address the need for a computationally efficient, extendable, reproducible and easy-to-use framework. ehrapy is compatible with major standards, such as Observational Medical Outcomes Partnership (OMOP), Common Data Model (CDM)^47^, HL7, FHIR or openEHR, with flexible support for common tabular data formats. Once loaded into an AnnData object, subsequent sharing of analysis results is made easy because AnnData objects can be stored and read platform independently. ehrapy’s rich documentation of the application programming interface (API) and extensive hands-on tutorials make EHR analysis accessible to both novices and experienced analysts.

As ehrapy remains under active development, users can expect ehrapy to continuously evolve. We are improving support for the joint analysis of EHR, genetics and molecular data where ehrapy serves as a bridge between the EHR and the omics communities. We further anticipate the generation of EHR-specific reference datasets, so-called atlases^98^, to enable query-to-reference mapping where new datasets get contextualized by transferring annotations from the reference to the new dataset. To promote the sharing and collective analysis of EHR data, we envision adapted versions of interactive single-cell data explorers, such as CELLxGENE^99^ or the UCSC Cell Browser^100^, for EHR data. Such web interfaces would also include disparity dashboards^20^ to unveil trends of preferential outcomes for distinct patient groups. Additional modules specifically for high-frequency time-series data, natural language processing and other data types are currently under development. With the widespread availability of code-generating large language models, frameworks such as ehrapy are becoming accessible to medical professionals without coding expertise who can leverage its analytical power directly. Therefore, ehrapy, together with a lively ecosystem of packages, has the potential to enhance the scientific discovery pipeline to shape the era of EHR analysis.

## Methods

All datasets that were used during the development of ehrapy and the use cases were used according to their terms of use as indicated by each provider.

### Design and implementation of ehrapy

A unified pipeline as provided by our ehrapy framework streamlines the analysis of EHR data by providing an efficient, standardized approach, which reduces the complexity and variability in data pre-processing and analysis. This consistency ensures reproducibility of results and facilitates collaboration and sharing within the research community. Additionally, the modular structure allows for easy extension and customization, enabling researchers to adapt the pipeline to their specific needs while building on a solid foundational framework.

ehrapy was designed from the ground up as an open-source effort with community support. The package, as well as all associated tutorials and dataset preparation scripts, are open source. Development takes place publicly on GitHub where the developers discuss feature requests and issues directly with users. This tight interaction between both groups ensures that we implement the most pressing needs to cater the most important use cases and can guide users when difficulties arise. The open-source nature, extensive documentation and modular structure of ehrapy are designed for other developers to build upon and extend ehrapy’s functionality where necessary. This allows us to focus ehrapy on the most important features to keep the number of dependencies to a minimum.

ehrapy was implemented in the Python programming language and builds upon numerous existing numerical and scientific open-source libraries, specifically matplotlib^101^, seaborn^102^, NumPy^103^, numba^104^, Scipy^105^, scikit-learn^53^ and Pandas^106^. Although taking considerable advantage of all packages implemented, ehrapy also shares the limitations of these libraries, such as a lack of GPU support or small performance losses due to the translation layer cost for operations between the Python interpreter and the lower-level C language for matrix operations. However, by building on very widely used open-source software, we ensure seamless integration and compatibility with a broad range of tools and platforms to promote community contributions. Additionally, by doing so, we enhance security by allowing a larger pool of developers to identify and address vulnerabilities^107^. All functions are grouped into task-specific modules whose implementation is complemented with additional dependencies.

### Data preparation

#### Dataloaders

ehrapy is compatible with any type of vectorized data, where vectorized refers to the data being stored in structured tables in either on-disk or database form. The input and output module of ehrapy provides readers for common formats, such as OMOP, CSV tables or SQL databases through Pandas. When reading in such datasets, the data are stored in the appropriate slots in a new AnnData^46^ object. ehrapy’s data module provides access to more than 20 public EHR datasets that feature diseases, including, but not limited to, Parkinson’s disease, breast cancer, chronic kidney disease and more. All dataloaders return AnnData objects to allow for immediate analysis.

#### AnnData for EHR data

Our framework required a versatile data structure capable of handling various matrix formats, including Numpy^103^ for general use cases and interoperability, Scipy^105^ sparse matrices for efficient storage, Dask^108^ matrices for larger-than-memory analysis and Awkward array^109^ for irregular time-series data. We needed a single data structure that not only stores data but also includes comprehensive annotations for thorough contextual analysis. It was essential for this structure to be widely used and supported, which ensures robustness and continual updates. Interoperability with other analytical packages was a key criterion to facilitate seamless integration within existing tools and workflows. Finally, the data structure had to support both in-memory operations and on-disk storage using formats such as HDF5 (ref. ^110^) and Zarr^111^, ensuring efficient handling and accessibility of large datasets and the ability to easily share them with collaborators.

All of these requirements are fulfilled by the AnnData format, which is a popular data structure in single-cell genomics. At its core, an AnnData object encapsulates diverse components, providing a holistic representation of data and metadata that are always aligned in dimensions and easily accessible. A data matrix (commonly referred to as ‘*X*’) stands as the foundational element, embodying the measured data. This matrix can be dense (as Numpy array), sparse (as Scipy sparse matrix) or ragged (as Awkward array) where dimensions do not align within the data matrix. The AnnData object can feature several such data matrices stored in ‘layers’. Examples of such layers can be unnormalized or unencoded data. These data matrices are complemented by an observations (commonly referred to as ‘obs’) segment where annotations on the level of patients or visits are stored. Patients’ age or sex, for instance, are often used as such annotations. The variables (commonly referred to as ‘var’) section complements the observations, offering supplementary details about the features in the dataset, such as missing data rates. The observation-specific matrices (commonly referred to as ‘obsm’) section extends the capabilities of the AnnData structure by allowing the incorporation of observation-specific matrices. These matrices can represent various types of information at the individual cell level, such as principal component analysis (PCA) results, t-distributed stochastic neighbor embedding (t-SNE) coordinates or other dimensionality reduction outputs. Analogously, AnnData features a variables-specific variables (commonly referred to as ‘varm’) component. The observation-specific pairwise relationships (commonly referred to as ‘obsp’) segment complements the ‘obsm’ section by accommodating observation-specific pairwise relationships. This can include connectivity matrices, indicating relationships between patients. The inclusion of an unstructured annotations (commonly referred to as ‘uns’) component further enhances flexibility. This segment accommodates unstructured annotations or arbitrary data that might not conform to the structured observations or variables categories. Any AnnData object can be stored on disk in h5ad or Zarr format to facilitate data exchange.

ehrapy natively interfaces with the scientific Python ecosystem via Pandas^112^ and Numpy^103^. The development of deep learning models for EHR data^113^ is further accelerated through compatibility with pathml^114^, a unified framework for whole-slide image analysis in pathology, and scvi-tools^115^, which provides data loaders for loading tensors from AnnData objects into PyTorch^116^ or Jax arrays^117^ to facilitate the development of generalizing foundational models for medical artificial intelligence^118^.

#### Feature annotation

After AnnData creation, any metadata can be mapped against ontologies using Bionty (https://github.com/laminlabs/bionty-base). Bionty provides access to the Human Phenotype, Phecodes, Phenotype and Trait, Drug, Mondo and Human Disease ontologies.

Key medical terms stored in an AnnData object in free text can be extracted using the Medical Concept Annotation Toolkit (MedCAT)^119^.

### Data processing

#### Cohort tracking

ehrapy provides a *CohortTracker* tool that traces all filtering steps applied to an associated AnnData object. To calculate cohort summary statistics, the implementation makes use of tableone^120^ and can subsequently be plotted as bar charts together with flow diagrams^121^ that visualize the order and reasoning of filtering operations.

#### Basic pre-processing and quality control

ehrapy encompasses a suite of functionalities for fundamental data processing that are adopted from scanpy^52^ but adapted to EHR data:

1. Regress out: To address unwanted sources of variation, a regression procedure is integrated, enhancing the dataset’s robustness.
2. Subsample: Selects a specified fraction of observations.
3. Balanced sample: Balances groups in the dataset by random oversampling or undersampling.
4. Highly variable features: The identification and annotation of highly variable features following the ‘highly variable genes’ function of scanpy is seamlessly incorporated, providing users with insights into pivotal elements influencing the dataset.

To identify and minimize quality issues, ehrapy provides several quality control functions:

1. Basic quality control: Determines the relative and absolute number of missing values per feature and per patient.
2. Winsorization: For data refinement, ehrapy implements a winsorization process, creating a version of the input array less susceptible to extreme values.
3. Feature clipping: Imposes limits on features to enhance dataset reliability.
4. Detect biases: Computes pairwise correlations between features, standardized mean differences for numeric features between groups of sensitive features, categorical feature value count differences between groups of sensitive features and feature importances when predicting a target variable.
5. Little’s MCAR test: Applies Little’s MCAR test whose null hypothesis is that data are MCAR. Rejecting the null hypothesis may not always mean that data are not MCAR, nor is accepting the null hypothesis a guarantee that data are MCAR. For more details, see Schouten et al.^122^.
6. Summarize features: Calculates statistical indicators per feature, including minimum, maximum and average values. This can be especially useful to reduce complex data with multiple measurements per feature per patient into sets of columns with single values.

Imputation is crucial in data analysis to address missing values, ensuring the completeness of datasets that can be required for specific algorithms. The ‘ehrapy’ pre-processing module offers a range of imputation techniques:

1. Explicit Impute: Replaces missing values, in either all columns or a user-specified subset, with a designated replacement value.
2. Simple Impute: Imputes missing values in numerical data using mean, median or the most frequent value, contributing to a more complete dataset.
3. KNN Impute: Uses *k*-nearest neighbor imputation to fill in missing values in the input AnnData object, preserving local data patterns.
4. MissForest Impute: Implements the MissForest strategy for imputing missing data, providing a robust approach for handling complex datasets.
5. MICE Impute: Applies the MICE algorithm for imputing data. This implementation is based on the miceforest (https://github.com/AnotherSamWilson/miceforest) package.

Data encoding can be required if categoricals are a part of the dataset to obtain numerical values only. Most algorithms in ehrapy are compatible only with numerical values. ehrapy offers two encoding algorithms based on scikit-learn^53^:

1. One-Hot Encoding: Transforms categorical variables into binary vectors, creating a binary feature for each category and capturing the presence or absence of each category in a concise representation.
2. Label Encoding: Assigns a unique numerical label to each category, facilitating the representation of categorical data as ordinal values and supporting algorithms that require numerical input.

To ensure that the distributions of the heterogeneous data are aligned, ehrapy offers several normalization procedures:

1. Log Normalization: Applies the natural logarithm function to the data, useful for handling skewed distributions and reducing the impact of outliers.
2. Max-Abs Normalization: Scales each feature by its maximum absolute value, ensuring that the maximum absolute value for each feature is 1.
3. Min-Max Normalization: Transforms the data to a specific range (commonly (0, 1)) by scaling each feature based on its minimum and maximum values.
4. Power Transformation Normalization: Applies a power transformation to make the data more Gaussian like, often useful for stabilizing variance and improving the performance of models sensitive to distributional assumptions.
5. Quantile Normalization: Aligns the distributions of multiple variables, ensuring that their quantiles match, which can be beneficial for comparing datasets or removing batch effects.
6. Robust Scaling Normalization: Scales data using the interquartile range, making it robust to outliers and suitable for datasets with extreme values.
7. Scaling Normalization: Standardizes data by subtracting the mean and dividing by the standard deviation, creating a distribution with a mean of 0 and a standard deviation of 1.
8. Offset to Positive Values: Shifts all values by a constant offset to make all values non-negative, with the lowest negative value becoming 0.

Dataset shifts can be corrected using the scanpy implementation of the ComBat^123^ algorithm, which employs a parametric and non-parametric empirical Bayes framework for adjusting data for batch effects that is robust to outliers.

Finally, a neighbors graph can be efficiently computed using scanpy’s implementation.

#### Embeddings

To obtain meaningful lower-dimensional embeddings that can subsequently be visualized and reused for downstream algorithms, ehrapy provides the following algorithms based on scanpy’s implementation:

1. t-SNE: Uses a probabilistic approach to embed high-dimensional data into a lower-dimensional space, emphasizing the preservation of local similarities and revealing clusters in the data.
2. UMAP: Embeds data points by modeling their local neighborhood relationships, offering an efficient and scalable technique that captures both global and local structures in high-dimensional data.
3. Force-Directed Graph Drawing: Uses a physical simulation to position nodes in a graph, with edges representing pairwise relationships, creating a visually meaningful representation that emphasizes connectedness and clustering in the data.
4. Diffusion Maps: Applies spectral methods to capture the intrinsic geometry of high-dimensional data by modeling diffusion processes, providing a way to uncover underlying structures and patterns.
5. Density Calculation in Embedding: Quantifies the density of observations within an embedding, considering conditions or groups, offering insights into the concentration of data points in different regions and aiding in the identification of densely populated areas.

#### Clustering

ehrapy further provides algorithms for clustering and trajectory inference based on scanpy:

1. Leiden Clustering: Uses the Leiden algorithm to cluster observations into groups, revealing distinct communities within the dataset with an emphasis on intra-cluster cohesion.
2. Hierarchical Clustering Dendrogram: Constructs a dendrogram through hierarchical clustering based on specified group by categories, illustrating the hierarchical relationships among observations and facilitating the exploration of structured patterns.

#### Feature ranking

ehrapy provides two ways of ranking feature contributions to clusters and target variables:

1. Statistical tests: To compare any obtained clusters to obtain marker features that are significantly different between the groups, ehrapy extends scanpy’s ‘rank genes groups’. The original implementation, which features a *t*-test for numerical data, is complemented by a *g*-test for categorical data.
2. Feature importance: Calculates feature rankings for a target variable using linear regression, support vector machine or random forest models from scikit-learn. ehrapy evaluates the relative importance of each predictor by fitting the model and extracting model-specific metrics, such as coefficients or feature importances.

#### Dataset integration

Based on scanpy’s ‘ingest’ function, ehrapy facilitates the integration of labels and embeddings from a well-annotated reference dataset into a new dataset, enabling the mapping of cluster annotations and spatial relationships for consistent comparative analysis. This process ensures harmonized clinical interpretations across datasets, especially useful when dealing with multiple experimental diseases or batches.

### Knowledge inference

#### Survival analysis

ehrapy’s implementation of survival analysis algorithms is based on lifelines^124^:

1. Ordinary Least Squares (OLS) Model: Creates a linear regression model using OLS from a specified formula and an AnnData object, allowing for the analysis of relationships between variables and observations.
2. Generalized Linear Model (GLM): Constructs a GLM from a given formula, distribution and AnnData, providing a versatile framework for modeling relationships with nonlinear data structures.
3. Kaplan–Meier: Fits the Kaplan–Meier curve to generate survival curves, offering a visual representation of the probability of survival over time in a dataset.
4. Cox Hazard Model: Constructs a Cox proportional hazards model using a specified formula and an AnnData object, enabling the analysis of survival data by modeling the hazard rates and their relationship to predictor variables.
5. Log-Rank Test: Calculates the *P* value for the log-rank test, comparing the survival functions of two groups, providing statistical significance for differences in survival distributions.
6. GLM Comparison: Given two fit GLMs, where the larger encompasses the parameter space of the smaller, this function returns the *P* value, indicating the significance of the larger model and adding explanatory power beyond the smaller model.

#### Trajectory inference

Trajectory inference is a computational approach that reconstructs and models the developmental paths and transitions within heterogeneous clinical data, providing insights into the temporal progression underlying complex systems. ehrapy offers several inbuilt algorithms for trajectory inference based on scanpy:

1. Diffusion Pseudotime: Infers the progression of observations by measuring geodesic distance along the graph, providing a pseudotime metric that represents the developmental trajectory within the dataset.
2. Partition-based Graph Abstraction (PAGA): Maps out the coarse-grained connectivity structures of complex manifolds using a partition-based approach, offering a comprehensive visualization of relationships in high-dimensional data and aiding in the identification of macroscopic connectivity patterns.

Because ehrapy is compatible with scverse, further trajectory inference-based algorithms, such as CellRank, can be seamlessly applied.

#### Causal inference

ehrapy’s causal inference module is based on ‘dowhy’^69^. It is based on four key steps that are all implemented in ehrapy:

1. Graphical Model Specification: Define a causal graphical model representing relationships between variables and potential causal effects.
2. Causal Effect Identification: Automatically identify whether a causal effect can be inferred from the given data, addressing confounding and selection bias.
3. Causal Effect Estimation: Employ automated tools to estimate causal effects, using methods such as matching, instrumental variables or regression.
4. Sensitivity Analysis and Testing: Perform sensitivity analysis to assess the robustness of causal inferences and conduct statistical testing to determine the significance of the estimated causal effects.

#### Patient stratification

ehrapy’s complete pipeline from pre-processing to the generation of lower-dimensional embeddings, clustering, statistical comparison between determined groups and more facilitates the stratification of patients.

### Visualization

ehrapy features an extensive visualization pipeline that is customizable and yet offers reasonable defaults. Almost every analysis function is matched with at least one visualization function that often shares the name but is available through the plotting module. For example, after importing ehrapy as ‘ep’, ‘ep.tl.umap(adata)’ runs the UMAP algorithm on an AnnData object, and ‘ep.pl.umap(adata)’ would then plot a scatter plot of the UMAP embedding.

ehrapy further offers a suite of more generally usable and modifiable plots:

1. Scatter Plot: Visualizes data points along observation or variable axes, offering insights into the distribution and relationships between individual data points.
2. Heatmap: Represents feature values in a grid, providing a comprehensive overview of the data’s structure and patterns.
3. Dot Plot: Displays count values of specified variables as dots, offering a clear depiction of the distribution of counts for each variable.
4. Filled Line Plot: Illustrates trends in data with filled lines, emphasizing variations in values over a specified axis.
5. Violin Plot: Presents the distribution of data through mirrored density plots, offering a concise view of the data’s spread.
6. Stacked Violin Plot: Combines multiple violin plots, stacked to allow for visual comparison of distributions across categories.
7. Group Mean Heatmap: Creates a heatmap displaying the mean count per group for each specified variable, providing insights into group-wise trends.
8. Hierarchically Clustered Heatmap: Uses hierarchical clustering to arrange data in a heatmap, revealing relationships and patterns among variables and observations.
9. Rankings Plot: Visualizes rankings within the data, offering a clear representation of the order and magnitude of values.
10. Dendrogram Plot: Plots a dendrogram of categories defined in a group by operation, illustrating hierarchical relationships within the dataset.

### Benchmarking ehrapy

We generated a subset of the UKB data selecting 261 features and 488,170 patient visits. We removed all features with missingness rates greater than 70%. To demonstrate speed and memory consumption for various scenarios, we subsampled the data to 20%, 30% and 50%. We ran a minimal ehrapy analysis pipeline on each of those subsets and the full data, including the calculation of quality control metrics, filtering of variables by a missingness threshold, nearest neighbor imputation, normalization, dimensionality reduction and clustering (Supplementary Table 1). We conducted our benchmark on a single CPU with eight threads and 60 GB of maximum memory.

ehrapy further provides out-of-core implementations using Dask^108^ for many algorithms in ehrapy, such as our normalization functions or our PCA implementation. Out-of-core computation refers to techniques that process data that do not fit entirely in memory, using disk storage to manage data overflow. This approach is crucial for handling large datasets without being constrained by system memory limits. Because the principal components get reused for other computationally expensive algorithms, such as the neighbors graph calculation, it effectively enables the analysis of very large datasets. We are currently working on supporting out-of-core computation for all computationally expensive algorithms in ehrapy.

We demonstrate the memory benefits in a hosted tutorial where the in-memory pipeline for 50,000 patients with 1,000 features required about 2 GB of memory, and the corresponding out-of-core implementation required less than 200 MB of memory.

The code for benchmarking is available at https://github.com/theislab/ehrapy-reproducibility. The implementation of ehrapy is accessible at https://github.com/theislab/ehrapy together with extensive API documentation and tutorials at https://ehrapy.readthedocs.io.

### PIC database analysis

#### Study design

We collected clinical data from the PIC^43^ version 1.1.0 database. PIC is a single-center, bilingual (English and Chinese) database hosting information of children admitted to critical care units at the Children’s Hospital of Zhejiang University School of Medicine in China. The requirement for individual patient consent was waived because the study did not impact clinical care, and all protected health information was de-identified. The database contains 13,499 distinct hospital admissions of 12,881 distinct pediatric patients. These patients were admitted to five ICU units with 119 total critical care beds—GICU, PICU, SICU, CICU and NICU—between 2010 and 2018. The mean age of the patients was 2.5 years, of whom 42.5% were female. The in-hospital mortality was 7.1%; the mean hospital stay was 17.6 d; the mean ICU stay was 9.3 d; and 468 (3.6%) patients were admitted multiple times. Demographics, diagnoses, doctors’ notes, laboratory and microbiology tests, prescriptions, fluid balances, vital signs and radiographics reports were collected from all patients. For more details, see the original publication of Zeng et al.^43^.

#### Study participants

Individuals older than 18 years were excluded from the study. We grouped the data into three distinct groups: ‘neonates’ (0–28 d of age; 2,968 patients), ‘infants’ (1–12 months of age; 4,876 patients) and ‘youths’ (13 months to 18 years of age; 6,097 patients). We primarily analyzed the ‘youths’ group with the discharge diagnosis ‘unspecified pneumonia’ (277 patients).

#### Data collection

The collected clinical data included demographics, laboratory and vital sign measurements, diagnoses, microbiology and medication information and mortality outcomes. The five-character English ICD-10 codes were used, whose values are based on the seven-character Chinese ICD-10 codes.

#### Dataset extraction and analysis

We downloaded the PIC database of version 1.1.0 from Physionet^1^ to obtain 17 CSV tables. Using Pandas, we selected all information with more than 50% coverage rate, including demographics and laboratory and vital sign measurements (Fig. 2). To reduce the amount of noise, we calculated and added only the minimum, maximum and average of all measurements that had multiple values per patient. Examination reports were removed because they describe only diagnostics and not detailed findings. All further diagnoses and microbiology and medication information were included into the observations slot to ensure that the data were not used for the calculation of embeddings but were still available for the analysis. This ensured that any calculated embedding would not be divided into treated and untreated groups but, rather, solely based on phenotypic features. We imputed all missing data through *k*-nearest neighbors imputation (*k* = 20) using the *knn_impute* function of ehrapy. Next, we log normalized the data with ehrapy using the *log_norm* function. Afterwards, we winsorized the data using ehrapy’s *winsorize* function to obtain 277 ICU visits (*n* = 265 patients) with 572 features. Of those 572 features, 254 were stored in the matrix *X* and the remaining 318 in the ‘obs’ slot in the AnnData object. For clustering and visualization purposes, we calculated 50 principal components using ehrapy’s *pca* function. The obtained principal component representation was then used to calculate a nearest neighbors graph using the *neighbors* function of ehrapy. The nearest neighbors graph then served as the basis for a UMAP embedding calculation using ehrapy’s *umap* function.

#### Patient stratification

We applied the community detection algorithm Leiden with resolution 0.6 on the nearest neighbor graph using ehrapy’s *leiden* function. The four obtained clusters served as input for two-sided *t*-tests for all numerical values and two-sided *g*-tests for all categorical values for all four clusters against the union of all three other clusters, respectively. This was conducted using ehrapy’s *rank_feature_groups* function, which also corrects *P* values for multiple testing with the Benjamini–Hochberg method^125^. We presented the four groups and the statistically significantly different features between the groups to two pediatricians who annotated the groups with labels.

Our determined groups can be confidently labeled owing to their distinct clinical profiles. Nevertheless, we could only take into account clinical features that were measured. Insightful features, such as lung function tests, are missing. Moreover, the feature representation of the time-series data is simplified, which can hide some nuances between the groups. Generally, deciding on a clustering resolution is difficult. However, more fine-grained clusters obtained via higher clustering resolutions may become too specific and not generalize well enough.

#### Kaplan–Meier survival analysis

We selected patients with up to 360 h of total stay for Kaplan–Meier survival analysis to ensure a sufficiently high number of participants. We proceeded with the AnnData object prepared as described in the ‘Patient stratification’ subsection to conduct Kaplan–Meier analysis among all four determined pneumonia groups using ehrapy’s *kmf* function. Significance was tested through ehrapy’s *test_kmf_logrank* function, which tests whether two Kaplan–Meier series are statistically significant, employing a chi-squared test statistic under the null hypothesis. Let *h*_*i*_*(t)* be the hazard ratio of group *i* at time *t* and *c* a constant that represents a proportional change in the hazard ratio between the two groups, then:

$${{\rm{H}}}_{{\rm{o}}}:{{\rm{h}}}_{1}({\rm{t}})={{\rm{h}}}_{2}({\rm{t}})$$

$${{\rm{H}}}_{{\rm{a}}}:{{\rm{h}}}_{1}({\rm{t}})={\rm{c}}* {{\rm{h}}}_{2}({\rm{t}}),{\rm{c}}\ne 1$$

This implicitly uses the log-rank weights. An additional Kaplan–Meier analysis was conducted for all children jointly concerning the liver markers AST, ALT and GGT. To determine whether measurements were inside or outside the norm range, we used reference ranges (Supplementary Table 2). *P* values less than 0.05 were labeled significant.

Our Kaplan–Meier curve analysis depends on the groups being well defined and shares the same limitations as the patient stratification. Additionally, the analysis is sensitive to the reference table where we selected limits that generalize well for the age ranges, but, due to children of different ages being examined, they may not necessarily be perfectly accurate for all children.

#### Causal effect of mechanism of action on LOS

Although the dataset was not initially intended for investigating causal effects of interventions, we adapted it for this purpose by focusing on the LOS in the ICU, measured in months, as the outcome variable. This choice aligns with the clinical aim of stabilizing patients sufficiently for ICU discharge. We constructed a causal graph to explore how different drug administrations could potentially reduce the LOS. Based on consultations with clinicians, we included several biomarkers of liver damage (AST, ALT and GGT) and inflammation (CRP and PCT) in our model. Patient age was also considered a relevant variable.

Because several different medications act by the same mechanisms, we grouped specific medications by their drug classes This grouping was achieved by cross-referencing the drugs listed in the dataset with DrugBank release 5.1 (ref. ^126^), using Levenshtein distances for partial string matching. After manual verification, we extracted the corresponding DrugBank categories, counted the number of features per category and compiled a list of commonly prescribed medications, as advised by clinicians. This approach facilitated the modeling of the causal graph depicted in Fig. 4, where an intervention is defined as the administration of at least one drug from a specified category.

Causal inference was then conducted with ehrapy’s ‘dowhy’^69^-based causal inference module using the expert-curated causal graph. Medication groups were designated as causal interventions, and the LOS was the outcome of interest. Linear regression served as the estimation method for analyzing these causal effects. We excluded four patients from the analysis owing to their notably long hospital stays exceeding 90 d, which were deemed outliers. To validate the robustness of our causal estimates, we incorporated several refutation methods:

- Placebo Treatment Refuter: This method involved replacing the treatment assignment with a placebo to test the effect of the treatment variable being null.
- Random Common Cause: A randomly generated variable was added to the data to assess the sensitivity of the causal estimate to the inclusion of potential unmeasured confounders.
- Data Subset Refuter: The stability of the causal estimate was tested across various random subsets of the data to ensure that the observed effects were not dependent on a specific subset.
- Add Unobserved Common Cause: This approach tested the effect of an omitted variable by adding a theoretically relevant unobserved confounder to the model, evaluating how much an unmeasured variable could influence the causal relationship.
- Dummy Outcome: Replaces the true outcome variable with a random variable. If the causal effect nullifies, it supports the validity of the original causal relationship, indicating that the outcome is not driven by random factors.
- Bootstrap Validation: Employs bootstrapping to generate multiple samples from the dataset, testing the consistency of the causal effect across these samples.

The selection of these refuters addresses a broad spectrum of potential biases and model sensitivities, including unobserved confounders and data dependencies. This comprehensive approach ensures robust verification of the causal analysis. Each refuter provides an orthogonal perspective, targeting specific vulnerabilities in causal analysis, which strengthens the overall credibility of the findings.

### UKB analysis

#### Study population

We used information from the UKB cohort, which includes 502,164 study participants from the general UK population without enrichment for specific diseases. The study involved the enrollment of individuals between 2006 and 2010 across 22 different assessment centers throughout the United Kingdom. The tracking of participants is still ongoing. Within the UKB dataset, metabolomics, proteomics and retinal optical coherence tomography data are available for a subset of individuals without any enrichment for specific diseases. Additionally, EHRs, questionnaire responses and other physical measures are available for almost everyone in the study. Furthermore, a variety of genotype information is available for nearly the entire cohort, including whole-genome sequencing, whole-exome sequencing, genotyping array data as well as imputed genotypes from the genotyping array^44^. Because only the latter two are available for download, and are sufficient for polygenic risk score calculation as performed here, we used the imputed genotypes in the present study. Participants visited the assessment center up to four times for additional and repeat measurements and completed additional online follow-up questionnaires.

In the present study, we restricted the analyses to data obtained from the initial assessment, including the blood draw, for obtaining the metabolomics data and the retinal imaging as well as physical measures. This restricts the study population to 33,521 individuals for whom all of these modalities are available. We have a clear study start point for each individual with the date of their initial assessment center visit. The study population has a mean age of 57 years, is 54% female and is censored at age 69 years on average; 4.7% experienced an incident myocardial infarction; and 8.1% have prevalent type 2 diabetes. The study population comes from six of the 22 assessment centers due to the retinal imaging being performed only at those.

#### Data collection

For the myocardial infarction endpoint definition, we relied on the first occurrence data available in the UKB, which compiles the first date that each diagnosis was recorded for a participant in a hospital in ICD-10 nomenclature. Subsequently, we mapped these data to phecodes and focused on phecode 404.1 for myocardial infarction.

The Framingham Risk Score was developed on data from 8,491 participants in the Framingham Heart Study to assess general cardiovascular risk^77^. It includes easily obtainable predictors and is, therefore, easily applicable in clinical practice, although newer and more specific risk scores exist and might be used more frequently. It includes age, sex, smoking behavior, blood pressure, total and low-density lipoprotein cholesterol as well as information on insulin, antihypertensive and cholesterol-lowering medications, all of which are routinely collected in the UKB and used in this study as the Framingham feature set.

The metabolomics data used in this study were obtained using proton NMR spectroscopy, a low-cost method with relatively low batch effects. It covers established clinical predictors, such as albumin and cholesterol, as well as a range of lipids, amino acids and carbohydrate-related metabolites.

The retinal optical coherence tomography–derived features were returned by researchers to the UKB^75,76^. They used the available scans and determined the macular volume, macular thickness, retinal pigment epithelium thickness, disc diameter, cup-to-disk ratio across different regions as well as the thickness between the inner nuclear layer and external limiting membrane, inner and outer photoreceptor segments and the retinal pigment epithelium across different regions. Furthermore, they determined a wide range of quality metrics for each scan, including the image quality score, minimum motion correlation and inner limiting membrane (ILM) indicator.

#### Data analysis

After exporting the data from the UKB, all timepoints were transformed into participant age entries. Only participants without prevalent myocardial infarction (relative to the first assessment center visit at which all data were collected) were included.

The data were pre-processed for retinal imaging and metabolomics subsets separately, to enable a clear analysis of missing data and allow for the *k*-nearest neighbors–based imputation (*k* = 20) of missing values when less than 10% were missing for a given participant. Otherwise, participants were dropped from the analyses. The imputed genotypes and Framingham analyses were available for almost every participant and, therefore, not imputed. Individuals without them were, instead, dropped from the analyses. Because genetic risk modeling poses entirely different methodological and computational challenges, we applied a published polygenic risk score for coronary heart disease using 6.6 million variants^73^. This was computed using the plink2 score option on the imputed genotypes available in the UKB.

UMAP embeddings were computed using default parameters on the full feature sets with ehrapy’s *umap* function. For all analyses, the same time-to-event and event-indicator columns were used. The event indicator is a Boolean variable indicating whether a myocardial infarction was observed for a study participant. The time to event is defined as the timespan between the start of the study, in this case the date of the first assessment center visit. Otherwise, it is the timespan from the start of the study to the start of censoring; in this case, this is set to the last date for which EHRs were available, unless a participant died, in which case the date of death is the start of censoring. Kaplan–Meier curves and Cox proportional hazards models were fit using ehrapy’s survival analysis module and the lifelines^124^ package’s Cox-PHFitter function with default parameters. For Cox proportional hazards models with multiple feature sets, individually imputed and quality-controlled feature sets were concatenated, and the model was fit on the resulting matrix. Models were evaluated using the C-index^127^ as a metric. It can be seen as an extension of the common area under the receiver operator characteristic score to time-to-event datasets, in which events are not observed for every sample and which ranges from 0.0 (entirely false) over 0.5 (random) to 1.0 (entirely correct). CIs for the C-index were computed based on bootstrapping by sampling 1,000 times with replacement from all computed partial hazards and computing the C-index over each of these samples. The percentiles at 2.5% and 97.5% then give the upper and lower confidence bound for the 95% CIs.

In all UKB analyses, the unit of study for a statistical test or predictive model is always an individual study participant.

The generalizability of the analysis is limited as the UK Biobank cohort may not represent the general population, with potential selection biases and underrepresentation of the different demographic groups. Additionally, by restricting analysis to initial assessment data and censoring based on the last available EHR or date of death, our analysis does not account for longitudinal changes and can introduce follow-up bias, especially if participants lost to follow-up have different risk profiles.

#### In-depth quality control of retina-derived features

A UMAP plot of the retina-derived features indicating the assessment centers shows a cluster of samples that lie somewhat outside the general population and mostly attended the Birmingham assessment center (Fig. 5b). To further investigate this, we performed Leiden clustering of resolution 0.3 (Extended Data Fig. 9a) and isolated this group in cluster 5. When comparing cluster 5 to the rest of the population in the retina-derived feature space, we noticed that many individuals in cluster 5 showed overall retinal pigment epithelium (RPE) thickness measures substantially elevated over the rest of the population in both eyes (Extended Data Fig. 9b), which is mostly a feature of this cluster (Extended Data Fig. 9c). To investigate potential confounding, we computed ratios between cluster 5 and the rest of the population over the ‘obs’ DataFrame containing the Framingham features, diabetes-related phecodes and genetic principal components. Out of the top and bottom five highest ratios observed, six are in genetic principal components, which are commonly used to represent genetic ancestry in a continuous space (Extended Data Fig. 9d). Additionally, diagnoses for type 1 and type 2 diabetes and antihypertensive use are enriched in cluster 5. Further investigating the ancestry, we computed log ratios for self-reported ancestries and absolute counts, which showed no robust enrichment and depletion effects.

A closer look at three quality control measures of the imaging pipeline revealed that cluster 5 was an outlier in terms of either image quality (Extended Data Fig. 9e) or minimum motion correlation (Extended Data Fig. 9f) and the ILM indicator (Extended Data Fig. 9g), all of which can be indicative of artifacts in image acquisition and downstream processing^128^. Subsequently, we excluded 301 individuals from cluster 5 from all analyses.

### COVID-19 chest-x-ray fate determination

#### Dataset overview

We used the public BrixIA COVID-19 dataset, which contains 192 chest x-ray images annotated with BrixIA scores^82^. Hereby, six regions were annotated by a senior radiologist with more than 20 years of experience and a junior radiologist with a disease severity score ranging from 0 to 3. A global score was determined as the sum of all of these regions and, therefore, ranges from 0 to 18 (S-Global). S-Global scores of 0 were classified as normal. Images that only had severity values up to 1 in all six regions were classified as mild. Images with severity values greater than or equal to 2, but a S-Global score of less than 7, were classified as moderate. All images that contained at least one 3 in any of the six regions with a S-Global score between 7 and 10 were classified as severe, and all remaining images with S-Global scores greater than 10 with at least one 3 were labeled critical. The dataset and instructions to download the images can be found at https://github.com/ieee8023/covid-chestxray-dataset.

#### Dataset extraction and analysis

We first resized all images to 224 × 224. Afterwards, the images underwent a random affine transformation that involved rotation, translation and scaling. The rotation angle was randomly selected from a range of −45° to 45°. The images were also subject to horizontal and vertical translation, with the maximum translation being 15% of the image size in either direction. Additionally, the images were scaled by a factor ranging from 0.85 to 1.15. The purpose of applying these transformations was to enhance the dataset and introduce variations, ultimately improving the robustness and generalization of the model.

To generate embeddings, we used a pre-trained DenseNet model with weights *densenet121-res224-all* of TorchXRayVision^129^. A DenseNet is a convolutional neural network that makes use of dense connections between layers (Dense Blocks) where all layers (with matching feature map sizes) directly connect with each other. To maintain a feed-forward nature, every layer in the DenseNet architecture receives supplementary inputs from all preceding layers and transmits its own feature maps to all subsequent layers. The model was trained on the *nih-pc-chex-mimic_ch-google-openi-rsna* dataset^130^.

Next, we calculated 50 principal components on the feature representation of the DenseNet model of all images using ehrapy’s *pca* function. The principal component representation served as input for a nearest neighbors graph calculation using ehrapy’s *neighbors* function. This graph served as the basis for the calculation of a UMAP embedding with three components that was finally visualized using ehrapy.

We randomly picked a root in the group of images that was labeled ‘Normal’. First, we calculated so-called pseudotime by fitting a trajectory through the calculated UMAP space using diffusion maps as implemented in ehrapy’s *dpt* function^57^. Each image’s pseudotime value represents its estimated position along this trajectory, serving as a proxy for its severity stage relative to others in the dataset. To determine fates, we employed CellRank^58,59^ with the *PseudotimeKernel*. This kernel computes transition probabilities for patient visits based on the connectivity of the *k*-nearest neighbors graph and the pseudotime values of patient visits, which resembles their progression through a process. Directionality is infused in the nearest neighbors graph in this process where the kernel either removes or downweights edges in the graph that contradict the directional flow of increasing pseudotime, thereby refining the graph to better reflect the developmental trajectory. We computed the transition matrix with a soft threshold scheme (Parameter of the *PseudotimeKernel*), which downweights edges that point against the direction of increasing pseudotime. Finally, we calculated a projection on top of the UMAP embedding with CellRank using the *plot_projection* function of the *PseudotimeKernel* that we subsequently plotted.

This analysis is limited by the small dataset of 192 chest x-ray images, which may affect the model’s generalizability and robustness. Annotation subjectivity from radiologists can further introduce variability in severity scores. Additionally, the random selection of a root from ‘Normal’ images can introduce bias in pseudotime calculations and subsequent analyses.

### Diabetes 130-US hospitals analysis

#### Study population

We used data from the Diabetes 130-US hospitals dataset that were collected between 1999 and 2008. It contains clinical care information at 130 hospitals and integrated delivery networks. The extracted database information pertains to hospital admissions specifically for patients diagnosed with diabetes. These encounters required a hospital stay ranging from 1 d to 14 d, during which both laboratory tests and medications were administered. The selection criteria focused exclusively on inpatient encounters with these defined characteristics. More specifically, we used a version that was curated by the Fairlearn team where the target variable ‘readmitted’ was binarized and a few features renamed or binned (https://fairlearn.org/main/user_guide/datasets/diabetes_hospital_data.html). The dataset contains 101,877 patient visits and 25 features. The dataset predominantly consists of White patients (74.8%), followed by African Americans (18.9%), with other racial groups, such as Hispanic, Asian and Unknown categories, comprising smaller percentages. Females make up a slight majority in the data at 53.8%, with males accounting for 46.2% and a negligible number of entries listed as unknown or invalid. A substantial majority of the patients are over 60 years of age (67.4%), whereas those aged 30–60 years represent 30.2%, and those 30 years or younger constitute just 2.5%.

#### Data analysis

All of the following descriptions start by loading the Fairlearn version of the Diabetes 130-US hospitals dataset using ehrapy’s dataloader as an AnnData object.

##### Selection and filtering bias

An overview of sensitive variables was generated using tableone. Subsequently, ehrapy’s *CohortTracker* was used to track the age, gender and race variables. The cohort was filtered for all Medicare recipients and subsequently plotted.

##### Surveillance bias

We plotted the HbA1c measurement ratios using ehrapy’s *catplot*.

##### Missing data and imputation bias

MCAR-type missing data for the number of medications variable (‘num_medications‘) were introduced by randomly setting 30% of the variables to be missing using Numpy’s *choice* function. We tested that the data are MCAR by applying ehrapy’s implementation of Little’s MCAR test, which returned a non-significant *P* value of 0.71. MAR data for the number of medications variable (‘num_medications‘) were introduced by scaling the ‘time_in_hospital’ variable to have a mean of 0 and a standard deviation of 1, adjusting these values by multiplying by 1.2 and subtracting 0.6 to influence overall missingness rate, and then using these values to generate MAR data in the ‘num_medications’ variable via a logistic transformation and binomial sampling. We verified that the newly introduced missing values are not MCAR with respect to the ‘time_in_hospital’ variable by applying ehrapy’s implementation of Little’s test, which was significant (0.01 × 10^−2^). The missing data were imputed using ehrapy’s mean imputation and MissForest implementation.

##### Algorithmic bias

Variables ‘race’, ‘gender’, ‘age’, ‘readmitted’, ‘readmit_binary’ and ‘discharge_disposition_id’ were moved to the ‘obs’ slot of the AnnData object to ensure that they were not used for model training. We built a binary label ‘readmit_30_days’ indicating whether a patient had been readmitted in fewer than 30 d. Next, we combined the ‘Asian’ and ‘Hispanic’ categories into a single ‘Other’ category within the ‘race’ column of our AnnData object and then filtered out and discarded any samples labeled as ‘Unknown/Invalid’ under the ‘gender‘ column and subsequently moved the ‘gender’ data to the variable matrix *X* of the AnnData object. All categorical variables got encoded. The data were split into train and test groups with a test size of 50%. The data were scaled, and a logistic regression model was trained using scikit-learn, which was also used to determine the balanced accuracy score. Fairlearn’s *MetricFrame* function was used to inspect the target model performance against the sensitive variable ‘race’. We subsequently fit Fairlearn’s *ThresholdOptimizer* using the logistic regression estimator with *balanced_accuracy_score* as the target object. The algorithmic demonstration of Fairlearn’s abilities on this dataset is shown here: https://github.com/fairlearn/talks/tree/main/2021_scipy_tutorial.

##### Normalization bias

We one-hot encoded all categorical variables with ehrapy using the *encode* function. We applied ehrapy’s implementation of scaling normalization with and without the ‘Age group’ variable as group key to scale the data jointly and separately using ehrapy’s *scale_norm* function.

### Reporting summary

Further information on research design is available in the Nature Portfolio Reporting Summary linked to this article.

## Online content

Any methods, additional references, Nature Portfolio reporting summaries, source data, extended data, supplementary information, acknowledgements, peer review information; details of author contributions and competing interests; and statements of data and code availability are available at 10.1038/s41591-024-03214-0.

## Supplementary information

Supplementary Tables 1 and 2

Reporting Summary

## Data Availability

Physionet provides access to the PIC database^43^ at https://physionet.org/content/picdb/1.1.0 for credentialed users. The BrixIA images^82^ are available at https://github.com/BrixIA/Brixia-score-COVID-19. The data used in this study were obtained from the UK Biobank^44^ (https://www.ukbiobank.ac.uk/). Access to the UK Biobank resource was granted under application number 49966. The data are available to researchers upon application to the UK Biobank in accordance with their data access policies and procedures. The Diabetes 130-US Hospitals dataset is available at https://archive.ics.uci.edu/dataset/296/diabetes+130-us+hospitals+for+years+1999-2008.

## Code availability

The ehrapy source code is available at https://github.com/theislab/ehrapy under an Apache 2.0 license. Further documentation, tutorials and examples are available at https://ehrapy.readthedocs.io. We are actively developing the software and invite contributions from the community.

Jupyter notebooks to reproduce our analysis and figures, including Conda environments that specify all versions, are available at https://github.com/theislab/ehrapy-reproducibility.
