# Supplementary material for: An open-source framework for end-to-end analysis of electronic health record data
Source: Nat Med. 2024 Sep 12;30(11):3369–80. doi: 10.1038/s41591-024-03214-0 (PMC11564094; doi:10.1038/s41591-024-03214-0)
Supplement: Supplementary file 1 — Supplementary Tables 1 and 2 [file 41591_2024_3214_MOESM1_ESM.pdf]

nature medicine

Supplementary information https://doi.org/10.1038/s41591-024-03214-0

An open-source framework for end-to-end analysis of electronic health record data

In the format provided by the authors and unedited

Supplementary Tables

Table 1

Dataset	Observations	Imputed variables	Mean missing [%]	Max memory [GB]	Runtime [minutes]
20% of UKB	97634	223	14.9	1.32	9.92
30% of UKB	146451	223	14.9	1.75	22.26
50% of UKB	244085	223	14.9	2.95	52.47
100% of UKB	488170	223	14.9	7.98	201.13

Table 1. Runtime and peak memory usage of an ehrapy pipeline on the UKB dataset.

Table 2

Measurement	Minimum	Maximum
Aspartate aminotransferase (AST)	10 IU/L	40 IU/L
Alanine transaminase (ALT)	10 IU/L	35 IU/L
Gamma-glutamyl transferase (GGT)	0 IU/L	35 IU/L

Table 2. Reference ranges of liver markers.
